# Estimating the effect of practicing nursing professionals density on cumulative carbapenem-resistance prevalence in gram-negative invasive Isolates: a 30 European country observational modeling study

**DOI:** 10.1186/s13756-022-01076-0

**Published:** 2022-02-22

**Authors:** Hani E. J. Kaba, Simone Scheithauer

**Affiliations:** Institute of Infection Control and Infectious Diseases, University Medical Center (UMG), Georg-August University Göttingen, Robert-Koch Str. 40, 37075 Göttingen, Germany

**Keywords:** Carbapenem-resistance, Gram-negatives, EARS-net, Surveillance, Nurse-density

## Abstract

**Background:**

The burden of antimicrobial-resistance, specifically carbapenem-resistance in gram-negative bacteria (CRGN), presents a serious public health threat worldwide. In Europe, Southern and Eastern countries (SEC) display a higher CRGN-prevalence as compared to Northern and Western countries (NWC). Since SEC also display lower nurse-density on average, we hypothesized that the occurrence of CRGN might correlate with nurse understaffing and therefore aimed at quantifying a potential independent effect of nurse-density on total CRGN in Europe.

**Methods:**

A 30-country cross-sectional study was conducted. Cumulative six-year CRGN-prevalence (2011–2016) in four gram-negative bacterial species was determined based on > 700 k clinical invasive isolates (EARS-net). We performed multivariable log-linear regression to provide estimations of the effect of nurse-density while adjusting to various health-system variables.

**Results:**

Multivariable analysis (adj.-R^2^ ~ 93%) suggested an average 0.4% [95%-CI 0.2–1.0%] CRGN-increase due to a decrement of one practicing nurse per week of hospital-stay of one population individual. Our modeling provided CRGN-estimations in two non-EARS-net countries (Switzerland and Turkey), which were almost equal to empirically estimated values (CAESAR-Network). Furthermore, a nurse-density-dependent moderation of the inter-species distribution balance was a likely pathway of the observed effect. These observations were specific for CRGN, in contrast to other resistance types in the same species.

**Conclusions:**

This is the first attempt of quantifying potential nurse-density effects on antimicrobial-resistance at national level. Our results suggest an increase in CRGN by decreasing nurse-density. Nurse-density is thus a novel factor that might improve our understanding of the unbalanced CRGN-distribution among sub-European regions. Consequently, integrating nurse-density in future AMR-policies could be beneficial.

**Supplementary Information:**

The online version contains supplementary material available at 10.1186/s13756-022-01076-0.

## Background

Resistance against antimicrobial agents (AMR) is a global public health threat, greatly contributing to the global burden of disease. AMR arises when microbial species survive exposure to one or more antimicrobial drugs which normally kill them or restrict their growth, rendering antimicrobial therapies against AMR pathogens ineffective. Hence, there is growing concern of a “post- antimicrobial era” and its threats to healthcare systems are of a global scale.

Inadequate antibiotic use, including overuse, misuse and substandard usage of antibiotics, triggers the onset of AMR [1, 2]. In Europe, AMR proportions significantly correlated with respective community antibiotic consumption levels [3, 4]. Moreover, seasonal fluctuations in consumption have been observed, revealing higher consumption in the south and east of Europe during the winter season [3]. Investigations found that antibiotic consumption had globally increased by 2015, especially in low- and middle-income countries, where the change in consumption was positively correlated with growth in national gross domestic products (GDP) [5]. Interestingly, countries with a higher overall regulatory level were found to have a significantly lower consumption of antibiotics. However, while regulatory measures might exist in countries with high consumption, they are either of voluntary character, or the level of compliance by healthcare professionals to such guidelines is low [6]. While antibiotic use significantly varies between countries, AMR prevalence is similarly variable. European AMR surveillance data suggested that countries in the south and east of Europe (SEC) display higher proportions of many AMR types compared to those located in the north and west of the continent (NWC) [7]. As compliance to prescription guidelines within distinct societies might involve a cultural aspect, an “out of the box” thinking approach has been suggested in past years, investigating the influence of cultural determinants on AMR. In this context, countries which apply more strict antibiotic stewardship (ABS) measures had lower proportions of methicillin-resistant *Staphylococcus aureus* (MRSA) compared to other countries, in which the implementation of ABS programs is less stringent [8]. Within this frame, pooled AMR proportions of several drug-species pairs were strongly associated with corruption [9], which can be considered as a cultural determinant of non-compliance to common guidelines. A strong association between corruption and antibiotic use strengthens this observation [10]. Recent research on the other hand, including our own work, suggested the association between climate (change) and AMR in both Europe and the USA [11–13].

The burden of infections with antibiotic-resistant bacteria was highest in countries known for low nurse-density, mostly in SEC in contrast to NWC [14, 15]. These countries are characterized by a health workforce pattern of low nurse-density and a relatively high density of physicians, compared to other European and OECD countries [16]. On the other hand, nurse understaffing was associated with an increased risk of acquiring healthcare associated infections (HCAI) [17]. Therefore, investigating the relationship between nurse-density and AMR prevalence, especially carbapenem-resistance in gram-negative bacterial species (CRGN), would be of high importance.

While a biologically plausible explanation for increased CRGN due to decreasing figures of nurses is missing at the first glance, such an association is expectedly moderated by other, health-system related factors, such as increased transmission of AMR-bacteria. One potential link between nurse understaffing and increased risk of transmission of carbapenem-resistant organisms, is presumably lower adherence to infection control practices, especially hand hygiene practices. However, while this argument is required for the establishment of such an association, it is not sufficient to explain it. The sufficient explanatory factor would be an increased transmission of carbapenem-resistant organisms at the expense of those who are non-carbapenem-resistant. Therefore, we hypothesized that nurse-density affects the distribution balance between the most common gram-negative bacteria (*Escherichia coli*, *Klebsiella pneumoniae*, *Pseudomonas aeruginosa* and *Acinetobacter* spp.). Selecting isolation frequency of invasive infectious pathogens as a surrogate indicator for the species-specific distribution, our aim was to quantify the independent impact of nurse-density on total CRGN-prevalence in a confounder-adjusted multivariable model.

## Methods

### Study design

We conducted a database observational study exploring the relationship between patterns of national AMR prevalence of four bacterial species (2011—2016) and nurse densities (2010–2015) in European countries.

### Study sample and units of observation

For species-specific distributions of resistance and isolation frequencies, the units of observation were invasive isolates. For bi- and multivariable analysis, the units of observation were national health systems, which are identical to the sovereign member states (n = 30) of the European Union and/ or the European Economic Area (EU/EEA) and participating in the EARS-net surveillance program. These were Austria, Belgium, Bulgaria, Croatia, Cyprus, Czechia, Denmark, Estonia, Finland, France, Germany, Greece, Hungary, Rep. Ireland, Italy, Latvia, Lithuania, Luxembourg, Malta, Netherlands, Poland, Portugal, Romania, Slovakia, Slovenia, Spain, Sweden, and the United Kingdom (all EU and EEA members as of 2016), in addition to Iceland and Norway (EEA members).

**Fig. 1:**
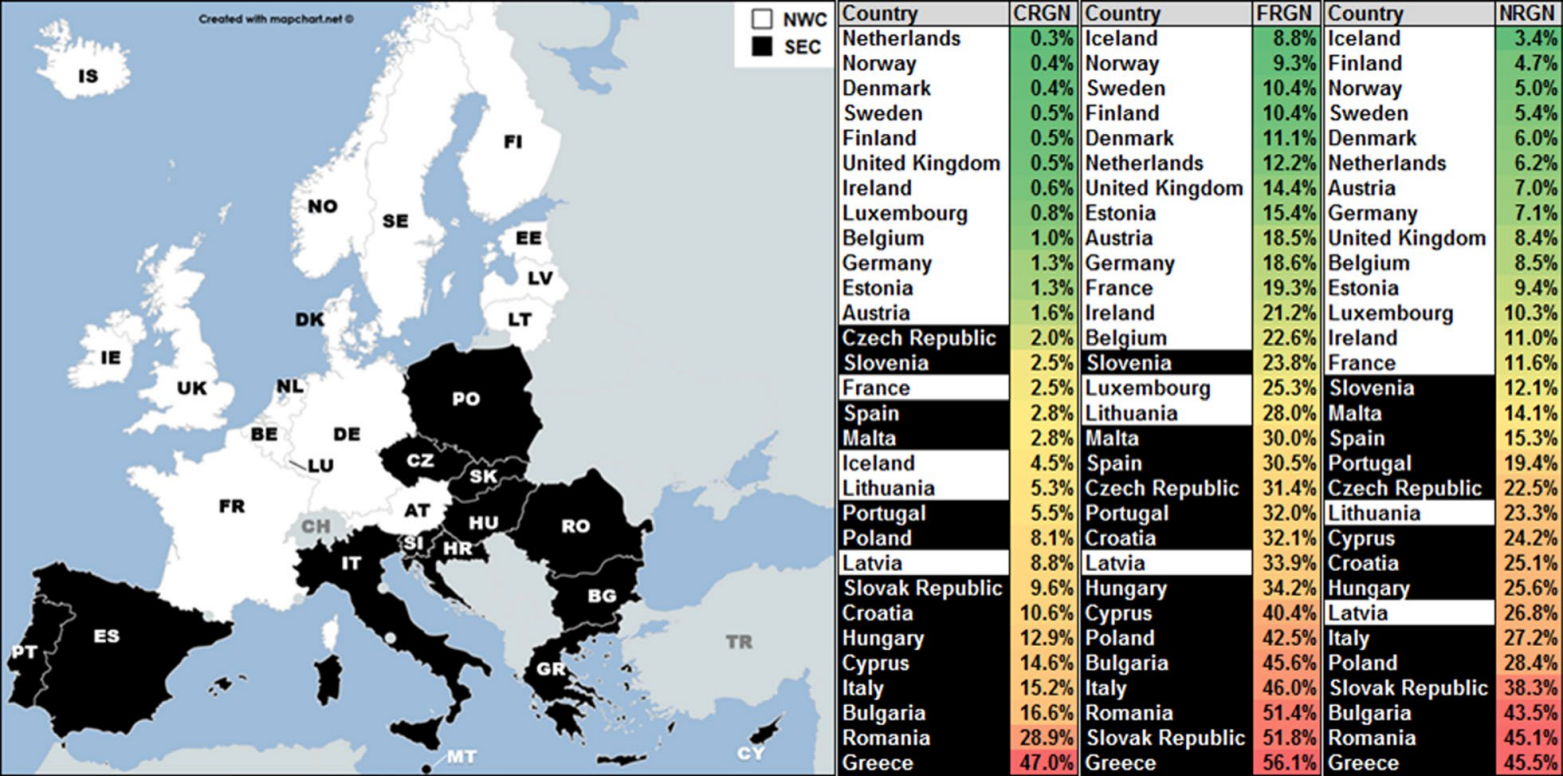
Country sample (left) and CRGN, FRGN and NRGN prevalence distribution (right). NWC = white, SEC = black

We further stratified this sample into two subgroups, based on the following criteria (a) geographic localization (Northern and Western countries = NWC; Southern and Eastern countries = SEC (Fig. 1; Additional file 1: Table S2), (b) status of nurse-density (low if density < EU/EEA median; otherwise high) and (c) status of physician-density (low if density < EU/EEA median; otherwise high).

### Dependent AMR variables

In 2017, the WHO published a priority list of antibiotic-resistant pathogens for which new antibiotics are urgently needed [18]. In accordance with this list, bacterial species-drug pairs of the priority 1 (critical) category were selected for analysis in this study. These were the carbapenem-resistant gram-negative bacteria *E. coli* (CREC), *K. pneumoniae* (CRKP), *P. aeruginosa* (CRPA) and *Acinetobacter* spp. (CRAc). As a control, we selected fluoroquinolone-resistant bacteria (FREC, FRKP, FRPA and FRAc respectively) and aminoglycoside-resistant bacteria (NREC, NRKP, NRPA and NRAc respectively) of the same species. Note that we preferred to use the more common term ‘species’ in order to refer to the respective bacterial identity, although in the case of *Acinetobacter* spp. no data of a single species was under observation, but rather aggregated data of the same genus.

The AMR surveillance data comprised invasive (blood and cerebrospinal fluid) isolates of antimicrobial susceptibility testing (AST), reported to the European Centre for Disease Prevention and Control (ECDC) by the 30 participating countries, which were in their turn provided by local laboratories in which the AST was performed. The antibiotic classes of interest were carbapenems (respective variable CRGN), fluoroquinolones (FRGN) and aminoglycosides (NRGN). We calculated the 6-year prevalence of each AMR species (2011—2016), in the same way as previously described [12]. The cumulative proportion of resistant bacteria for each class, was obtained by calculating the sum of all resistant isolates across years (2011–2016) and bacterial species for each country (∑R), and the sum of total reported isolates tested for the respective class across years (2011–2016) and bacterial species for each country (∑B), and then by dividing ∑R by ∑B. We thereby treated all four gram-negative bacterial species (*E. coli*, *K. pneumoniae*, *P. aeruginosa* and *Acinetobacter* spp.) as a single entity.

### Independent variable nurse-density

We selected practicing nursing professionals, i. e. according to the ISCO 08 group definitions, code 2221, those who “… treat and provide care for people who are physically or mentally ill, the elderly, the injured or physically or mentally disabled …” [19]. This would include all nursing professionals regardless of the type of healthcare setting (inpatient and outpatient settings). Data were retrieved from two databases: Eurostat and OECD and the final variable values were calculated same as the variable ‘docs’ (physicians) [12]. The unit of this variable (nurses) was practicing nursing professionals per 1000 population. For detailed description, consult Additional file 1, Section ‘Predictors and confounders’, point 3 and Additional file 1: Tables S3a–d). For the density of nursing professionals employed in hospitals, which also includes midwives (nurses_H), consult point 4 of the same section.

### Potential confounders

We attempted to adjust for a number of confounders in our modelling approach that were either known, or expected to be associated with AMR. These were physician density (docs), antibiotic consumption in humans (DDD), the corruption perception index (CPI), health expenditure (hsp) and curative care beds (beds). These variables were created in our previous work [12]. Additionally, we considered total disability-adjusted life years (DALYs) as a national indicator on total burden of disease, the average in-patient length of stay (ALOS) as well as figures on isolation frequencies of each bacterial species, processed from the same source of AMR data (EARS-net). Details on data processing and calculations of all potential confounder variables can be found in the Additional file 1 in section’Predictors and Confounders’.

### Isolation frequency data

We used the proportion of each bacterial species within all reported gram-negative isolates (EARS-net, 2013–2016) as an indicator of the isolation frequency of invasive infections due to the respective species. This variable can be thought of being a surrogate for the species-specific distribution among the pool of gram-negative bacteria that cause or may cause invasive infections, in both the inpatient and outpatient sectors (endogenous or exogenous transmissions), which are preventable (in contrast to community associated transmissions). They also might include gram-negative bacteria isolated from contaminated blood cultures. However it has been observed that the species studied herein (Enterobacteriaceae and *P*. *aeruginosa*) represent true bacteremia in > 95% of the cases when isolated from blood cultures [20]. Consequently, the differences in proportions between the species describe the isolation frequency balance between them. Note that nurse-staffing figures were negatively associated with the incidence of positive blood cultures in healthcare settings [21]. Consult ‘Discussion’ section for further discussion of this approach and Additional file 1, section ‘Predictors and confounders’, points 12 and 13 as well as Additional file 1: Table S1 for detailed information on the variables used.

### Multivariable analysis

All modeling steps were performed as previously described, including model validation and evaluation [12]. Diagnostic models (e.g. MRSA, FRGN and NRGN) were neither validated nor interpreted. Note that for multivariable regression analysis, Acpr had to undergo log-transformation (natural logarithm) due to a suspected minimal violation of nonlinearity with log_CRGN (Additional file 1: Fig. S1b). We compared nurse-density dependent ΔCRGN estimations between each log-level model (Acpr in M1.S, M2.S and M3.S) and its corresponding log–log model (log_Acpr in M1.M, M2.M and M3.M). The log–log models resulted from substituting Acpr by log_Acpr in the log-level models. Potentially outlying cases of high concern were identified by calculating Cook’s distance. All multivariable analysis were performed using IBM SPSS Statistics 25–26. Consult Additional file 1, section ‘Multivariable analysis’ for additional information on all modeling steps.

### Additional files

Supplementary material, including detailed information on methods, data collection and processing as well as supplementary figures/ tables is provided in Additional file 1.

## Results

### Descriptive statistics of AMR in gram-negative invasive isolates

We observed aggregated gram-negative invasive isolates from 30 EU/EEA countries between 2011 and 2016. In total, test results on 711,090 invasive isolates (*E. coli*, *K. pneumoniae*, *P. aeruginosa* and *Acinetobacter* spp. combined) for carbapenem-resistance, 735,094 isolates for fluoroquinolone-resistance and 726,134 for aminoglycoside-resistance have been reported. Different species-specific proportions for each class were obtained (data was restricted to the interval 2013—2016 due to inconsistent *Acinetobacter* spp. reporting before 2013). The proportion of carbapenem-resistance was highest in *Acinetobacter* spp. (52.0% [95%-CI 51.2–52.7%]) followed by *P. aeruginosa* (18.5% [18.2–18.9%]), *K. pneumoniae* (7.1% [6.9–7.2%]) and *E. coli* (0.08% [0.07–0.09%]). Fluoroquinolone-resistance was similarly highest in *Acinetobacter* spp. (57.7% [57.0–58.4%]) followed by *K. pneumoniae* (28.9% [28.6–29.2%]), *E. coli* (21.1% [21.0–21.3%]) and *P. aeruginosa* (19.4% [19.0–19.7%]). Finally, aminoglycoside-resistance was also highest in *Acinetobacter* spp. (50.9% [50.2–51.6%]) followed by *K. pneumoniae* (24.0% [23.7–24.2%]), *P. aeruginosa* (14.0% [13.7–14.3%]) and *E. coli* (9.7% [9.6–9.8%]).

We created a six-year cumulative carbapenem-resistance prevalence variable (CRGN) of all four species as a single national index for each country during the observed interval (2011—2016). CRGN ranged from 0.3% in the Netherlands to 47% in Greece (Fig. 1) and showed tenfold higher proportions in SEC as compared to NWC on average (p < 0.001, Table 1b).

**Table 1:** Descriptive statistics: means of the AMR and health system variables

	n	CRGN	FRGN	NRGN	nurses	docs	DDD	CPI	hsp	DALYs	beds	ALOS
a. EU/EEA												
Yes	30	2.6%	26.6%	13.1%	6.6	3.6	20.3	253	52.8	27,462	358	7.6
b. geo												
NWC	16	0.9%	17.0%	7.8%	9.7	3.6	16.9	313	57.6	26,232	342	7.4
SEC	14	10.1%	37.3%	25.4%	5.0	3.4	22.1	209	45.6	29,666	407	7.6
p (2-tailed test)		p < 0.001	p < 0.001	p < 0.001	p < 0.001	0.608	0.038	p < 0.001	0.025	0.110	0.448	0.400
c. nurses_2G												
Low	15	9.6%	34.2%	25.6%	4.9	3.4	20.9	214	44.7	30,579	374	7.6
High	15	1.0%	18.6%	7.1%	9.8	3.7	18.5	317	59.4	25,637	335	7.1
p (2-tailed test)		p < 0.001	p < 0.001	p < 0.001	p < 0.001	0.436	0.389	p < 0.001	0.003	0.011	0.775	0.412
d. docs_2G												
Low	15	2.8%	30.0%	14.1%	5.5	3.1	22.6	239	44.5	27,462	368	7.8
High	15	2.0%	21.2%	11.0%	7.8	4.1	17.9	279	54.6	26,880	358	7.4
p (2-tailed test)		0.653	0.285	0.345	0.119	p < 0.001	0.110	0.491	0.102	0.642	0.897	0.217

Stratifications: (a) EU/EEA sample, (b) NWC/ SEC (variable geo), (c) countries according to nurses density (low = true if value < EU/EEA median; otherwise false), (d) countries according to physicians density (low = true if value < EU/EEA median; otherwise false). Abbreviations and variable descriptions (proportion of gram-negative isolates in %): CRGN = carbapenem-resistant gram-negative bacteria, FRGN = fluoroquinolone-resistant gram-negative bacteria, NRGN = aminoglycoside-resistant gram-negative bacteria, nurses = nurse density [nurses per 1000 population], docs = physicians density [physicians per 1000 population], DDD = total antimicrobial consume in the community[doses per day and 1000 population], CPI = corruption perception index [points], hsp = health spending [% of GDP], DALYs = all causes disability adjusted life years (rounded, [per 10^5^ population]), beds = acute care beds density (rounded, [per 10^5^ population]), ALOS = average length of stay in hospitals [days]. The Mann–Whitney test was used for determining statistical significance of differences between subgroups. Statistical significance was assumed when p < 0.05

### SEC displayed a twofold lower density of practicing nursing professionals than NWC on average

We estimated the density of practicing nursing professionals (nurses) in EU/EEA countries (2010—2015) and compared the distribution of this variable between NWC and SEC. Furthermore, we characterized other health system indicators of NWC and SEC in order to identify variables with potential significant differences (Table 1a–d). In particular, we compared physician-density (docs), acute care bed density (ac_beds), standardized total burden of disease (DALYs) and average inpatient length of stay (ALOS) values. SEC showed an almost twofold lower nurse-density on average compared to NWC (p < 0.001), while no significant difference was found for any other variable mentioned above (Table 1b). Indeed, belonging to the SEC group strongly coincided with having a sub EU/EEA-median (i. e. “low”) nurse density (OR = 26.00, p < 0.001, n = 30). Accordingly, CRGN displayed distribution patterns between countries with low (< EU/EEA median) and high (≥ EU/EEA median) nurse density that were almost identical to those of SEC and NWC respectively (Table 1c). In contrast, no significant differences were observed upon comparing CRGN levels between countries with low (< EU/EEA median) and high (≥ EU/EEA median) physician-density (Table 1d).

### Nurse-density moderates the effect of inter-species isolation balance on CRGN in adjusted multivariable models

**Fig. 2:**
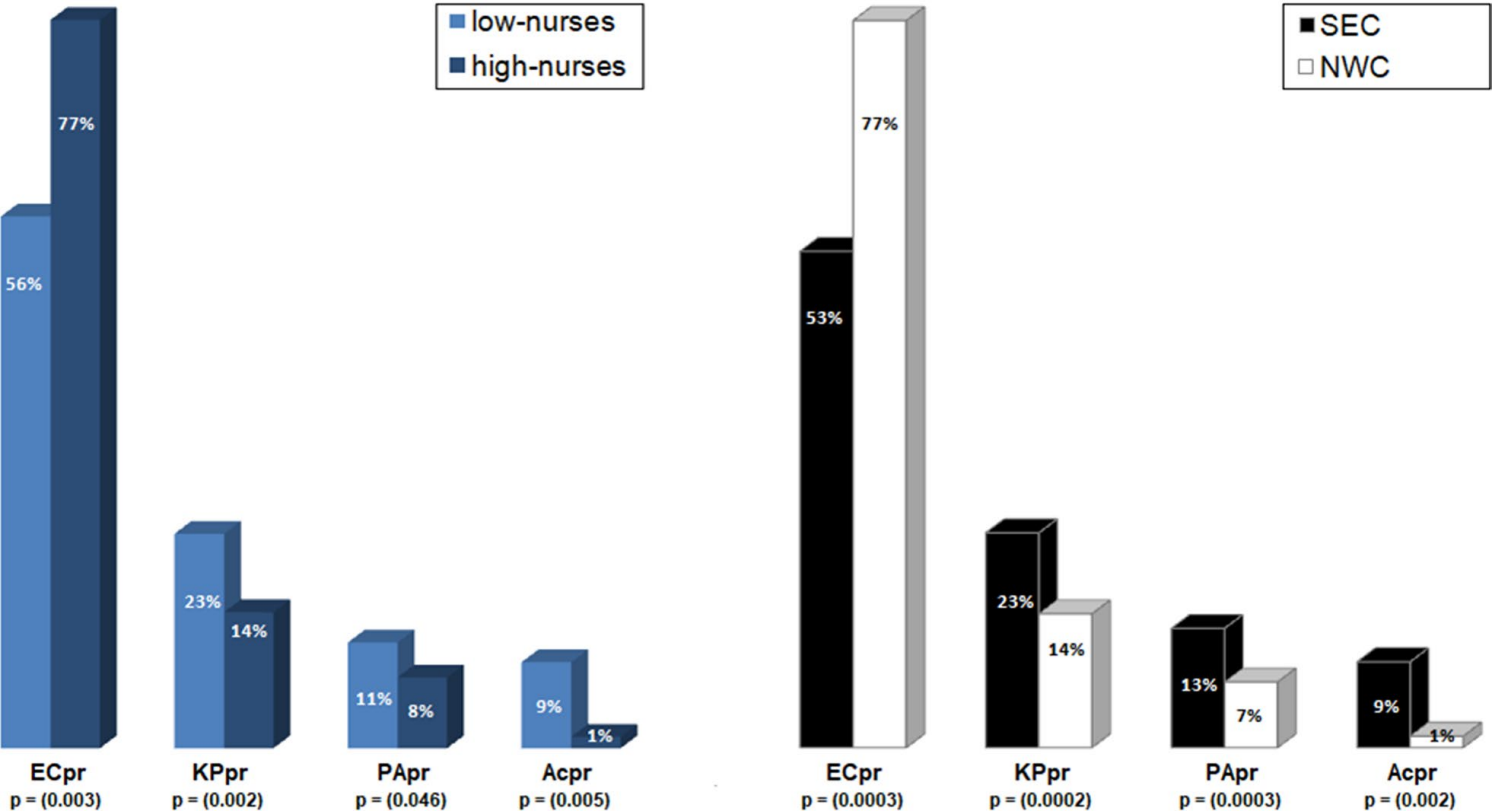
Medians of species-specific proportions between countries with low / high nurse-density (left) and NWC / SEC (right). Statistical significance (MW-Test) is given when p < 0.05

Since a potential CRGN-nurse-density association is rather indirect, a pathway that might explain such an association (if present) could be the interference of nurse availability in the transmission frequency balance between carbapenem-resistant and non-carbapenem-resistant bacteria. Given the different species-specific carbapenem-resistance proportions between isolates of the different bacterial species as seen above, we calculated the species-specific proportion of each bacterial type among all reported gram-negative isolates (2013—2016). These were ECpr for *E. coli*, KPpr for *K. pneumoniae*, PApr for *P. aeruginosa* and Acpr for *Acinetobacter* spp. We used these proportions as indicators of each species-specific isolation frequency (and thus of healthcare setting related transmission events with invasive infections). Interestingly, we found lower *E. coli* but higher *K*. *pneumoniae*, *P*. *aeruginosa* and *Acinetobacter* spp. isolation frequencies of invasive bacteria in low nurse-density countries (low nurses) as compared to high nurses countries. The same phenomenon was observed when these proportions were compared between NWC and SEC (Fig. 2).

When multivariable regression was run in EU/EEA using all potential independent variables, nurse-density was an independent predictor of CRGN alongside Acpr, CPI and DDD, yielding M1.S (adj.-R^2^ = 92%). Consequently, a decrease of one practicing nursing professional per 1000 population was associated with an 8.3% [95%-CI 1.0–16.2%] increase (p = 0.027) in CRGN on average, ceteris paribus (cp.).

**Fig. 3:**
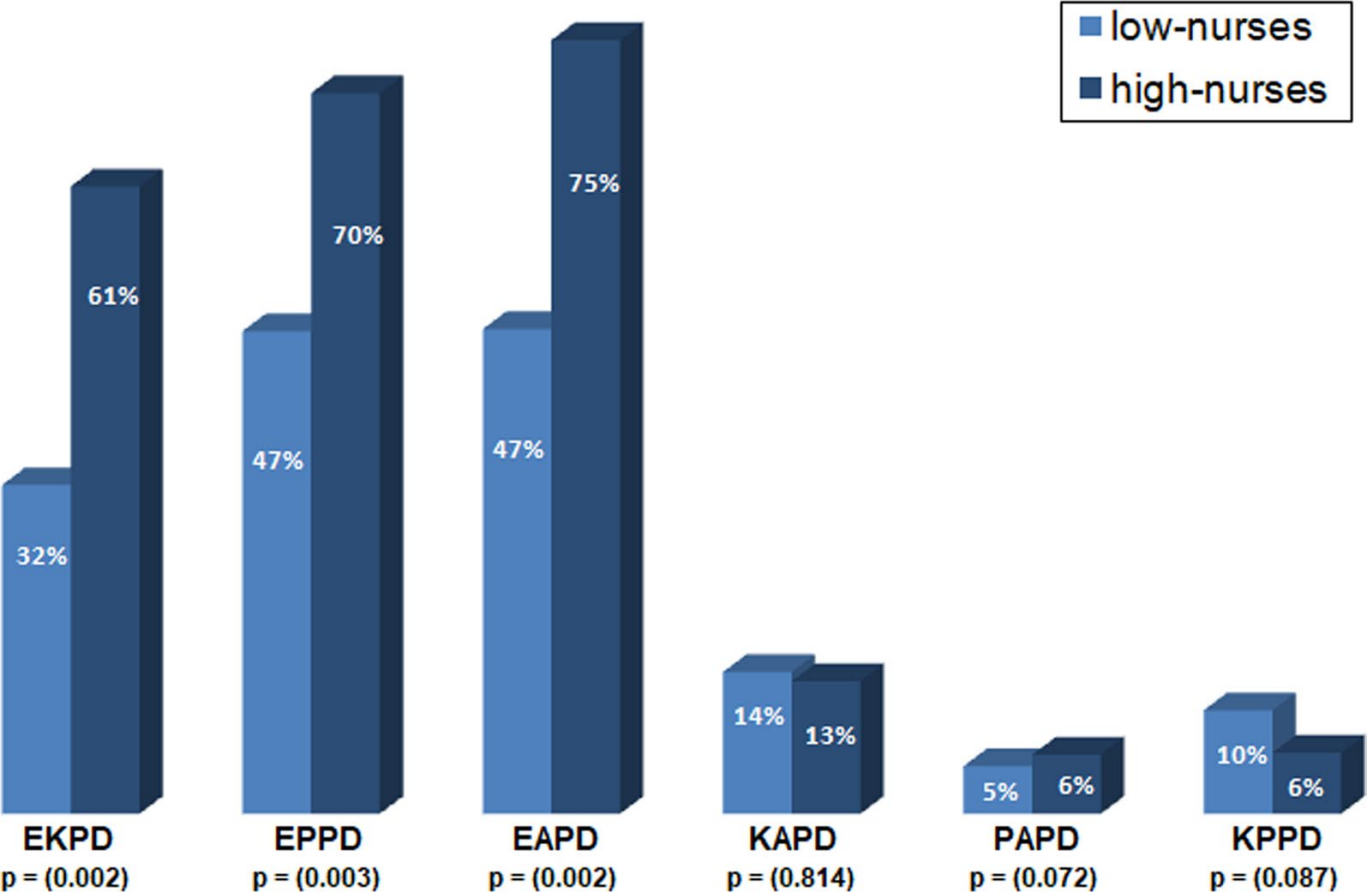
Medians of differences in species-specific proportions of all species combinations between countries with low / high nurse-density. Statistical significance (MW-Test) is given when p < 0.05

To test the hypothesis of interference in the isolation frequency balance, we created variables that encode the difference in proportions between all possible species combinations. As expected, only combinations that included *E. coli* (EKPD for *K*. *pneumoniae*, EPPD for *P*. *aeruginosa* and EAPD for *Acinetobacter* spp.) showed significant lower values in low nurse-density countries as compared to those with high nurse-density (Fig. 3). This means that the isolation frequencies of *E. coli* and any other species were closer to each other in low nurse density countries, indicating a higher probability for positive non-*E. coli* culture events at the expense of positive *E. coli* cultures. We entered each proportion difference and the respective interaction term with nurse-density (intX; with X being any of EKPD, EPPD or EAPD) into model M1.S and selected for independent factors. Only intEAPD (interaction term of EAPD with nurse-density) was retained as an independent factor by the model. Remarkably, no independent main effects of EAPD nor of nurse-density were observed. According to the resulting model, M2.S (adj-R^2^ = 94%), and considering that EAPD is dimensionless, a decrease of one practicing nursing professional per 1000 population associated with a 13.3% [95%-CI 4.7–22.6%] increase (p = 0.003) in CRGN-prevalence on average, cp.

**Fig. 4:**
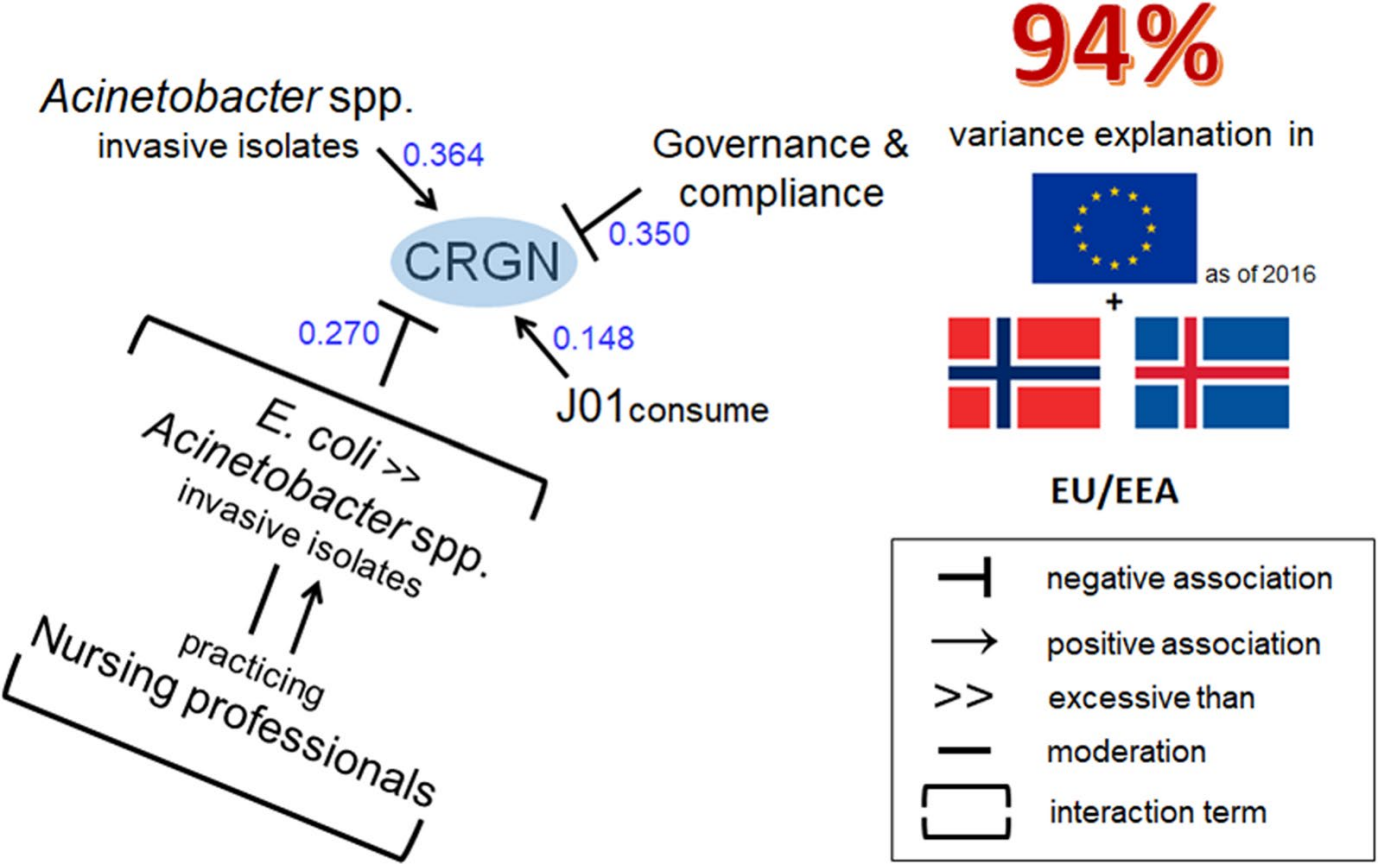
Summary of model M2.M and the contribution of different factors to CRGN prevalence (standardized regression coefficients, shown in blue)

Note that Acpr had to undergo log-transformation due to suspected non-linearity with log_CRGN. Substituting Acpr by log_Acpr in M1.S and M2.S yielded the log–log models M1.M and M2.M respectively (Table 2, Fig. 4).

**Table 2:** Summary of the multivariate log–log models obtained in this study

Model	adj.-R^2^ (%)	Method	Dependent variable	ß_0_		Nurses	DDD	CPI	log_Acpr	intEAPD	intEAlos	intEAlos_H
M1.M	93.5	Step-wise forward selection	log_CRGN (n = 30)	0.238^Ø^	b	− 0.085^*^	0.030^*^	− 0.007^**^	0.549^***^			
					ß	− 0.221	0.133	− 0.321	0.462			
M2.M	93.8	Step-wise forward selection	log_CRGN (n = 30)	− 0.150^**^	b		0.034^**^	− 0.008^***^	0.432^***^	− 0.114^**^		
					ß		0.148	− 0.350	0.364	− 0.270		
M3.M	92.7	Forced entry	log_CRGN (n = 30)	0.100^Ø^	b		0.032^*^	− 0.009^***^	0.494^***^		− 0.519^*^	
					ß		0.141	− 0.382	0.416		− 0.202	
M4.M	93.3	Forced entry	log_CRGN (n = 27)	0.567^Ø^	b		0.033^*^	− 0.011^***^	0.443^***^			− 0.730^*^
					ß		0.145	− 0.477	0.372			− 0.156

adj.-R^2^: adjusted coefficient of determination, ß_0_: intercept, b: regression coefficient, ß: standardized regression coefficient. Only variables with significant regression coefficients were retained by the respective model through step-wise forward selection (inclusion cut-off p < 0.05; exclusion cut-off p ≥ 0.10) unless otherwise indicated. Ø denotes p > 0.05, * denotes p < 0.05, ** denotes p < 0.01, *** denotes p < 0.001. Information on log-linear models M1.S, M2.S and M3.S are provided in the Supplementary Material, section ‘Model equations’

In the next step, we standardized intEAPD by the national average length of hospital stay (ALOS) and used the resulting variable, dubbed intEAlos, as a substitute for intEAPD in M2.M, yielding M3.M. According to this model (adj.-R^2^ = 93%), a decrement of 1 practicing nursing professional per week of hospital stay of a random population individual was associated with a 0.4% [95%-CI 0.1–0.6%] increase (p = 0.011) in CRGN on average, cp. This corresponds to an average 68% [95%-CI 14–148%] increase in CRGN due to a decrease of 1 nurse per 1000 aggregated days of hospital stay of random population individuals (Table 2).

The nurse-density related variables mentioned above represent nurse figures throughout each respective health system, regardless of the sector of their occupational activity, as nurses’ influence on AMR might exceed bedside tasks. In order to specifically investigate the relationship between CRGN and the density of nursing professionals (including midwives) in hospitals, we substituted the nurses variable in intEAlos by a hospital-specific nurses-density variable (nurses_H), yielding M4.M. According to this model (adj.-R2 = 93%), a decrement of 1 practicing nursing professional per week of hospital stay of a random population individual was associated with a 0.51% [95%-CI 0.01–1.02%] increase (p = 0.046) in CRGN on average, cp. (Table 2).

### Model validation and evaluation

We compared nurse-density dependent ΔCRGN estimations between each log-level model (M.1S, M2.S and M3.S) and its corresponding log–log model (M1.M, M2.M and M3.M respectively) and those were statistically indifferent due to overlapping confidence intervals (Additional file 1: Table S4). Models M1.M, M2.M and M3.M (Table 2) complied with all assumptions of linear regression (a–i presented in the Additional file 1), with only substantial concerns (consult Additional file 1: Figures S1–S7 and Tables S5–S6). No influential cases of high concern were observed (Additional file 1: Table S7).

Next, we evaluated the robustness of M3.M in estimating national CRGN-prevalence both within and outside EU/EEA. Given its relatively large goodness of fit (adj.-R^2^ ~ 93%), almost all empirically estimated national CRGN-prevalence values fell within the M3.M estimated 95% confidence interval for each respective EU/EEA country (Table 3). We compared the M3.M estimation of CRGN in Switzerland and Turkey (both outside EU/EEA) with empirically estimated CRGN in those countries (CAESAR Network, WHO European Region, 2013—2016). The fitted estimations for both countries were relatively close to empirical values, falling within the respective confidence intervals of M3.M (Switzerland: 0.9% vs. 0.4% [95%-CI 0.2–1.0%]; Turkey: 31% vs. 36% [15–90%], Table 3).

**Table 3:** Predictive power of M3.M in EU/EEA, Switzerland and Turkey

Country	Code	CRGN (%)	PRE_CRGN [CI 95%]
Austria	AT	1.6	1.1% [0.5–2.5%]
Belgium	BE	1.0	0.9% [0.4–2.0%]
Bulgaria	BG	16.6	21.8% [9.6–49.2%]
Croatia	HR	10.6	11.8% [5.4–25.9%]
Cyprus	CY	14.6	10.3% [4.6–23.4%]
Czech Republic	CZ	2.0	4.0% [1.8–9.0%]
Denmark	DK	0.4	0.5% [0.2–1.1%]
Estonia	EE	1.3	0.9% [0.4–2.1%]
Finland	FI	0.5	0.5% [0.2–1.2%]
France	FR	2.5	2.4% [1.1–5.4%]
Germany	DE	1.3	1.1% [0.5–2.5%]
Greece	GR	47.0	37.5% [16.2–86.9%]
Hungary	HU	12.9	9.2% [4.1–20.6%]
Iceland	IS	4.5	4.9% [2.0–12.0%]
Ireland	IE	0.6	1.2% [0.5–2.8%]
Italy	IT	15.2	13.4% [6–30.1%]
Latvia	LV	8.8	8.4% [3.7–19.3%]
Lithuania	LT	5.3	4.9% [2.2–10.7%]
Luxembourg	LU	0.8	1.0% [0.4–2.2%]
Malta	MT	2.8	3.6% [1.6–7.8%]
Netherlands	NL	0.3	0.4% [0.2–0.9%]
Norway	NO	0.4	0.2% [0.1–0.6%]
Poland	PL	8.1	7.3% [3.3–15.9%]
Portugal	PT	5.5	3.4% [1.6–7.4%]
Romania	RO	28.9	27.2% [12.1–61.4%]
Slovak Republic	SK	9.6	10.9% [5–23.9%]
Slovenia	SI	2.5	3.0% [1.3–6.6%]
Spain	ES	2.8	2.1% [0.9–4.7%]
Sweden	SE	0.5	0.3% [0.1–0.7%]
United Kingdom	UK	0.5^■^	1.4% [0.6–3.1%]
Switzerland	CH	0.9	0.4% [0.2–1.0%]
Turkey	TR	30.5	36.3% [14.7–89.8%]

Estimated national cumulative carbapenem resistance proportion (PRE_CRGN) by model M3.M (adj. R^2^ = 93%) compared to empirical national proportion (CRGN). ^■^ denotes empirical proportion not covered by M3.M estimation (95% CI)

### Moderation effects of nurse-density were specific for CRGN in contrast to FRGN and NRGN

The next question we asked was whether the significant independent effects obtained by M1.M and M3.M also explain prevalence variance of other resistance types in the same bacterial species. We thus calculated the 6-year cumulative fluoroquinolone (FRGN) and aminoglycoside (NRGN) prevalence indices (separately, Fig. 1) in analogy to CRGN. Both variables displayed the unbalanced geographical distribution observed for CRGN with significantly higher proportions in SEC as well as in low nurse-density countries (Table 1b and 1c). We repeated the previous steps leading to M1.M and M3.M with either FRGN or NRGN as dependent variables (log-transformed). In multivariable regression, neither the FRGN (M4.S), nor the NRGN (M5.S) model did retain nurse-density as a significant independent factor. Similarly, neither the FRGN (M6.S), nor the NRGN (M7.S) model displayed an independent moderation effect of EAPD by nurse-density (intEAlos). Taken together, these results indicate that the observed effects of nurse-density on the resistance prevalence in gram-negative invasive isolates were specific for CRGN under the applied test conditions (Additional file 1, model Eqs. 4—7). Similar results were obtained when intEAPD was divided by (i) average hospital discharges or (ii) total DALYs, where the significant regression coefficients were specific for CRGN, but neither for FRGN nor NRGN (all dependent variables log-transformed, Additional file 1, model Eqs. 9—14).

In order to check for potential species-independent random effects in the models, we modeled log_MRSA [12] with the independent variables of M3.M. As expected, intEAlos had no significant contribution to log_MRSA variance explanation (M8.S, Additional file 1, model Eq. 8).

### Nurse-density effects on single-species carbapenem-resistant prevalence within CRGN

Finally, we investigated whether nurse-density explains parts of the carbapenem-resistance prevalence variance of the single CRGN species (excluding *E. coli*). As expected, no changes occurred to the model *P. aeruginosa* (log_CRPA) obtained in our previous work [12] since nurse-density was not retained by the model (p = 0.140, M15.S, Additional file 1, model Eq. 15). Similar observation was made for *K. pneumoniae* (log_CRKP, p = 0.248, M16.S, Additional file 1, model Eq. 16). In contrast, nurse-density contributed to variance explanation carbapenem-resistant *Acinetobacter* spp. (log_CRAc) with a negative association observed similar to M1.M/S (p = 0.031, M17.S, Additional file 1, model Eq. 17). Note that two countries were excluded from CRAc and one from CRKP analysis upon log-transformation, due to empirical zero prevalence data.

## Discussion

Our models suggest that national nurse-density influences CRGN in clinical invasive isolates by moderating the inter-species distribution balance among gram-negative bacteria. This is most likely achieved through the degree of basic hygiene compliance [22]. This effect was independent of antibiotic consumption, corruption perception and *Acinetobacter* spp. isolates proportion. The length of hospital stay standardized derivate of these models suggests an average 0.4% increase for every week of stay of a random population individual and this increase can rise up to 2.5-fold (upper end of 95%-CI) for hypothetically aggregated 1000 days of hospital stay (M3.M). These results are hardly comparable with the existing literature, due to the novelty of our work. However, the composition of the models seems to be plausible and scholarly debate of human healthcare resources with consideration of health system and service delivery characteristics might provide some useful insights on relationships.

*Escherichia coli* is the most frequent cause of bloodstream infections in high-income countries, such as EU/EEA [23], which complies with our observation of ECpr displaying the highest proportion in our sample. We found that countries with low nurse-density displayed lower ECpr and higher proportions of the other isolated species on average, which translates into a smaller proportion difference between ECpr and any of the other species in those countries. However, only EAPD, i.e. the proportion difference between *E. coli* and *Acinetobacter* spp. was associated with CRGN in interaction with nurse-density. This interaction suggests that the effect of EAPD on CRGN is different for different values of nurse-density. However, the reason for the association with EAPD in contrast to EKPD (proportion difference with *K. pneumoniae*) or EPPD (proportion difference with *P. aeruginosa*) remains elusive. A possible explanation involves three phenomena. First, nurse understaffing was associated with increased risk of HCAI [17], which might be mediated by increased workload [24]. For instance, non-compliance with relevant hand hygiene guidelines could to some extent be a result of high work overload [22]. Second, multidrug-resistant *Acinetobacter* spp. are highly persistent in the patient environment (e.g. bed rails, supply carts) and more frequently transmitted via contaminated gowns, gloves or hands of HCWs than other bacteria, with HCW status of a nurse identified as an independent risk factor for such a contamination [25, 26]. Finally, the contrast in carbapenem-resistance probability of a random isolate is comparably very large between the two species under focus, *Acinetobacter* spp. and *E. coli* (~ 52% vs. 0.1% respectively). Taken together, these phenomena plausibly explain to some degree the pathway that connects nurse understaffing with increased carbapenem-resistance proportion at population level.

We have built our estimations based on models, where we have attempted to adjust for independent factors known to influence AMR. Antibiotic consumption in humans and indicators on corruption and governance (e.g. CPI) have been previously identified as strong independent factors of different AMR types or factors driving AMR [3, 4, 9, 10, 12, 27]. Our present models are no exception, which strengthens their validity and explanatory power. Indeed, models like M3.M display > 93% of explained CRGN variance and performed well in estimating CRGN levels in two countries that have not been included in the sample.

An interesting observation was the specificity of associations to carbapenem-resistance, in contrast to other resistance types in the same bacterial species (fluoroquinolone and aminoglycoside resistance). While no definite explanation for this observation can be made, this could be due to the contrast in carbapenem-resistance probabilities of random isolates between *Acinetobacter* spp. and *E. coli*. Given the fact that most *A. baumanii* (the most common *Acinetobacter* spp.) strains are intrinsically resistant to at least two classes of antibiotics [28]; strains with combined resistance are transmitted with a very high probability leading to an increased prevalence of isolates that are more difficult to treat.

Another interesting observation was that reducing the analysis to nurses employed in the hospital sector delivered identical results. This confirms that nurses’ availability in hospitals is most crucial to reduce CRGN augmentation.

In total, our results suggest that nurse-density influences carbapenem-resistance prevalence figures in European countries. Therefore, this variable contributes to some extent to total variance explanation of CRGN and helps to reduce the gap of understanding regional disparities in CRGN distribution among European countries. These results also point towards a potential role of nurse availability in preventing resistance spread. Although some explanations make a mediation by bedside practices plausible, it cannot be excluded that the observed effects could be mediated by other factors, such as the involvement of nurses (upon non-deficiency of nurses), in stewardship programs. However, the data at hand is not suitable to test such hypotheses.

One major limitation of the study is the comparison of national data. Neither AMR, nor e.g. the use of antibiotics, is national. The reality is that sub-national variations within single health systems exist. However, it was our intention to investigate whether national health system characteristics are associated with AMR in adjusted multivariable models, specifically nurse-density, which might help to understand differences in AMR distributions across Europe. Sub-regional comparison from European countries is furthermore difficult given the lack of reliable regional data from many EU/EEA countries. The main limitation might derive from variations in reporting nurse figures between countries and across the years. The numbers are highly dependent on the reliability and standardization of the sources. Missing values on national “practicing” nursing professionals were substituted by values of “professionally active” of each health system, potentially leading to overestimation of nurse-density in those countries. Both NWC (France, Rep. Ireland and the Netherlands) as well as SEC (Portugal and Slovakia) were affected by this limitation. Furthermore, some countries, in particular Austria, Greece and the UK, reported figures of nurses practicing only in hospitals, potentially leading to underestimation of the figures.

Another limitation of our study was that bacterial isolates were restricted to invasive infections and do not contain e.g. samples of urinary tract, superficial skin infections or bacterial colonization of mucosal surfaces. Since treatment regimens of patients with bloodstream infections (BSIs) is strongly linked to the frequency with which blood cultures are taken, variations in blood culture frequency may occur causing underestimation of the resistance prevalence in countries with low blood culture frequency. Simultaneously, therapeutic failure/ non-response would increase the frequency of blood culture sampling, which might lead to an overestimation of the resistance prevalence [28]. These limitations would have an impact on how well the isolation frequencies reported herein would represent transmission frequencies of the different bacterial species. Furthermore, the number of reporting laboratories from each country is variable, potentially leading to some bias in the data source. We utilized the currently best available data source. Additionally, data on antibiotic consumption in humans was not coherent, with some countries reporting total healthcare data including the hospital sector, while the majority reported data of the primary care sector only. However, the risk of overestimating primary care consumption in those countries should be low, as primary care antibiotic consumption on average accounts for 90% of the total consumption [4]. The use of a carbapenem specific (J01DH) consumption variable might have been more accurate to estimate carbapenem-resistance in ecological studies rather than a universal antibiotic (J01) consumption variable. Including J01DH was not possible due to a high missing proportion in the country sample. Finally, while we used a complex selection of various indicators, some important factors were missing from the analysis. Most notably antibiotic use in the animal sector, due to missing and inconsistent country data for the observation period. The inclusion of other factors might have added explanatory value to the models or on the other hand, altered their composition.

## Conclusions

In general, nurse understaffing (in addition to lower education levels) is associated with poorer care [29]. Part of this are increased transmissions of persistent bacteria, causing infections, which are difficult to treat. Since SEC and low nurse-density countries display almost exactly equal characteristics, we suggest the density of practicing nursing professionals as a factor that helps to understand the north-to-south and west-to-east increasing resistance gradients. Our results open the door to further research on the roles of nurses in reducing AMR prevalence levels. Furthermore, they encourage considering the role of nurses in fighting AMR spread in future health policies, since this role has been largely overlooked in policy papers and medical recommendations so far.

For a full list of variable names and description, please consult Additional file 1: Table S1 in the Supplementary Material.

## Supplementary Information

**Additional file 1.** Supplementary Material.

## Data Availability

The datasets supporting the conclusions of this article are included within the article and its additional files. All raw data were extracted from public databases through the information (accession codes, dates) indicated the Supplementary Material (Additional file 1: Table S1 and Reference List). Information on study design, data collection, variable values, processing of variables and sub-variables, modeling steps, transformations and validation procedures are also provided in the Supplementary Material.

## Abbreviations

SEC: Southern and Eastern European countries (see Additional file 1: Table S2 and Fig. 1)
NWC: Northern and Western European countries (see Additional file 1: Table S2 and Fig. 1)
EU/EEA: European Union and European Economic Area
AMR: Antimicrobial resistance
CRGN: Carbapenem-resistant gram-negatives
FRGN: Fluoroquinolone–resistant gram-negatives
NRGN: Aminoglycoside-resistant gram-negatives
HCAI: Healthcare associated infections
HCW: Healthcare workforce
