# Supplementary material for: Estimating the effect of practicing nursing professionals density on cumulative carbapenem-resistance prevalence in gram-negative invasive Isolates: a 30 European country observational modeling study
Source: Antimicrob Resist Infect Control. 2022 Feb 22;11:41. doi: 10.1186/s13756-022-01076-0 (PMC8862581; doi:10.1186/s13756-022-01076-0)
Supplement: Supplementary file 1 — Additional file 1. Supplementary Material. [file 13756_2022_1076_MOESM1_ESM.docx]

**Supplementary Material**

**Estimating the effect of practicing nursing professionals density on cumulative carbapenem-resistance prevalence in gram-negative invasive isolates – A 30 European country observational modeling study**

Hani E. J. Kaba and Simone Scheithauer

Institute of Infection Control and Infectious Diseases, University Medical Center, Georg August University Göttingen, Germany.

[hani.kaba@med.uni-goettingen.de](mailto:hani.kaba@med.uni-goettingen.de)

**Data collection and processing**

For data collection, a Microsoft^®^ Excel master file was used to store collected data. Collection of data underwent a quality control by double checking data values. Datasets were extracted and updated from each source between March 2018 and September 2019. Sub-variables are variables of a given indicator, whose values are processed to yield the final value of the variable which is included in pair-wise or multivariate analysis. Each value of a sub-variable was extracted from the source at least on two different occasions. The equality of such values was compared using the IF(condition;then;otherwise) function in Microsoft^®^ Excel. When the same sub-variable was found to have two unequal values from each round of extraction, its value was determined by a further round of extraction. The same IF function was also used to avoid errors resulting from changes in the order of listing countries, where the country lists of different variables, sub-variables and extraction round were compared. Basic variables are sub-variables that are further processed to yield additional sub-variables, e.g. by multiplying (for interaction terms) or subtraction (for differences in proportion; see below Supplementary Tables S1 and S2). All calculations were performed with Microsoft^®^ Excel unless otherwise indicated. Log-transformations, bi- as well as multivariable analysis and confidence intervals were calculated using IBM SPSS Statistics 25 – 26 (IBM Corporation, Armonk, NY, USA) unless otherwise indicated. Calculations of odds ratios, including tests for statistical significance, were performed with both programs separately. SPSS IBM Statistics 25 – 27, Microsoft^®^ Excel and Microsoft^®^ PowerPoint were used to create diagrams/ bar plots.

**AMR data**

AMR data were retrieved from EARS-Net for the period between 2011 and 2016. These data are exclusively based on invasive isolates (from blood or cerebrospinal fluid samples). According to the source “… this restriction prevents some of the inconsistencies that arise from differences in clinical case definitions, different sampling frames or heterogeneous healthcare utilization that would otherwise confound the data analysis if isolates from all anatomical sites were accepted” [1]. It is worth to note that invasive isolates may not be representative of isolates of the same bacterial species from other type of infections, e.g. urinary tract, pneumonia, skin or wound infections. Variable values are presented in Supplementary Table S2).

**Predictors and confounders**

We attempted to adjust for the following confounders in our modelling approach (variable values are presented in Supplementary Table S3a):

1. Consumption of antibacterials for systemic use (ATC group J01): initially, we intended to collect combined community (i.e. primary care) and hospital antimicrobial use. Due to missing information in hospital data for 10 countries (> 25%), we restricted the data collection on the primary care sector. Some countries, including Romania and Cyprus, largely reported combined data (primary care + hospital sectors). However, there is little risk of overestimating the primary care consumption in these countries, because the primary care sector accounts for ca. 90% of the antibiotic consumption [2]. Furthermore, it is worth to note that the provided data were inhomogeneous, as they contained either sales or reimbursement values.

Carbapenem antibiotics (J01DH) are typically reserve antibiotics for treatment of resistant infections, which are mainly consumed in the hospital sector. Data on carbapenem consumption were incomplete; information was missing for most of the observation period (2010 - 2015) at the time of analysis. Therefore, we obtained data on antibiotic consumption in humans (antibiotics for systemic use, J01) from ESAC-net (ECDC), for the years 2010 - 2015 (i.e. with a time lag of 1 year compared to AMR data). Standardized consumption is given as DDD (daily defined dose per 1000 individuals and day) following WHO recommendations.

1. Physician density: the total number of physicians provided for each country by Eurostat (hlth_rs_phys) was divided for each year of observation (2010 - 2015) by the country’s population for the respective year (demo_pjan). The yearly values were then multiplied by 1000 before means were calculated, as missing values existed (e.g. for Cyprus and Bulgaria, information on 4 years was missing). This resulted in the mean density of physicians per 1000 population, which constitutes the values of the respective variable.
2. Nurse-density: regarding nurse numbers, different descriptions of professions exist between the different countries in our sample. Our analysis was not intended to be restricted to one healthcare sector. Also, our analysis was not restricted to nurses who predominantly work at the bedside, but was rather intended to include those involved in e.g. (citation) “…the supervision of other health care workers, working … in the practical application of preventive … measures, … education and other health promotion activities, … conducting research” [3]. According to the WHO, examples for professions belonging to this category are professional nurse, specialist nurse, nurse practitioner, clinical nurse, district nurse, operating theatre nurse, public health nurse, nurse anaesthetist or nurse educator [4]. Thus, we have chosen this professional category because it is the broadest category that comprises most (if not all) nursing professions, which require formal education at (higher) nursing institutes. Eurostat (HLTH_RS_PRSNS) provided information distinguishing between “nurses” and “nursing professionals” including or excluding midwives and other caring personnel, then further stratified into those professionally active, licensed to practice or practicing. The difference is explained by Eurostat [5] (citation):

- “practising, in other words, health care professionals providing services directly to patients;
- professionally active, in other words, ‘practising’ professionals plus health care professionals for whom their medical education is a prerequisite for the execution of their job;
- licensed, in other words, health care professionals who are registered and entitled to practise as health care professionals”.

We excluded midwives and other caring personnel (according to the source) and selected “practicing nursing professionals” as a basis for calculating nurse-density. According to the ISCO-08 group definitions, code 2221, nursing professionals are those who “… treat and provide care for people who are physically or mentally ill, the elderly, the injured or physically or mentally disabled …”. This would include all nursing professionals regardless of the type of healthcare setting (inpatient and outpatient settings). The Eurostat [5] and OECD definitions [6] of the numbers presented in their database is in frame of these definitions. For these reasons, we selected variables representing “practicing” or “professionally active” nursing professionals. However, none of the databases provided complete data for the time interval under observation.

No data were available in Eurostat for the following countries: Belgium, Czechia, France, Rep. of Ireland, Latvia, Netherlands, Portugal and Slovakia. Missing information for all those countries were complemented by data retrieved from the OECD database, where the figures corresponded to the density of “professionally active” nurses per 1000 population for France, Rep. Ireland, the Netherlands, Portugal, Slovakia and to the density of “practicing” nurses for the other countries. Further variation was observed since Austria, Greece and the UK provided data only on nursing professionals working in hospitals.

For all countries, means of yearly figures were calculated to obtain the mean nurse-density per 1000 population, which constitutes the values of the respective independent variable, which we dubbed as (nurses). Eurostat data was processed as indicated above for physician-density. Population data (demo_pjan) for the respective years was also retrieved from Eurostat. Finally, both databases (Eurostat and OECD) were merged following the rule, that OECD data was included only when the information was missing in the Eurostat database (as of 15 August 2018). For each given country, the yearly values derived either from Eurostat or from OECD. No mixing of yearly values within cases was allowed. The two datasets (Eurostat and OECD) showed a good correlation coefficient (rs = 0.787, p = 0.0002, n = 17). Reversing the rule mentioned above during merging (i.e. Eurostat data was included only when the information was missing in the OECD database, as of 15 August 2018) did neither change the significance of the intEAlos regression coefficient in M3.M (see M18.S, model equation 18 below), nor the interpretation of the fold-change in log_CRGN (Supplementary Table S4). Consult Supplementary Tables S3a-d for information on the numbers extracted from each database (in the case of Eurostat processed numbers), as well as the final values of the nurse-density variable (nurses, Table S3a) and the alternative nurse-density variable (alt_nurses, Table S3d).

In summary, two major databases which include data on nurse density of EU/EEA (i.e. EARS-net participating) countries exist. Both of which contain completely missing data for distinct countries during the observation period. Merging both databases restores completeness of data. Complementing missing values of either database with values from the other leads to a significant contribution of intEAlos to log_CRGN variance explanation, which is the main message of this study.

1. Density of nursing professionals and midwives employed in hospital: the total number of nursing professionals and midwives employed in hospitals (estimated) provided for each country by Eurostat (HLTH_RS_PRSHP1) was divided for each year of observation (2011 - 2015) by the country’s population for the respective year (demo_pjan). The yearly values were then multiplied by 1000 before means were calculated. This resulted in the mean density per 1000 population, which constitutes the values of the respective variable (nurses_H). Data for three countries (Czechia, Slovakia and Sweden) were completely missing, reducing the sample size to n = 27 whenever this variable was used in analysis. For three countries (Austria, Greece and UK), the values of nurses and nurses_H are technically identical because these countries exclusively reported hospital-employed nurses figures. The only difference was due to the different observation periods (2010-2015 and 2011-2015 respectively). Interestingly, Finland displayed a nurses_H that was close to 10% of the nurses variable (Supplementary Table S3a). This might be due to the organization of the Finnish healthcare system, in which primary care is delivered in municipal healthcare centers [7] and/ or the problem of Finnish nurses largely seeking career opportunities outside the healthcare sector [8].
2. The Corruption Perceptions Index (CPI) (Transparency International) is an indicator on poor governance and corruption. CPI was given in points on a scale from 0 to 100. For each country, CPI scores were calculated by summing up the points scored by each country between 2012 and 2015.
3. Health expenditure: we preferred the use of health spending (hsp) as % of GDP instead of the Gross Domestic Product (GDP), which is a general indicator of economic wealth of a country rather than reflecting the size of the healthcare economy of a given country. On the other hand, hsp measures the final consumption of healthcare goods and services and is therefore health system specific, in contrast to GDP. Data were retrieved for all countries from the World Bank database (indicator: SH.XPD.CHEX.GD.ZS). The score of hsp was calculated as the sum of all values from 2010 to 2015, since no missing yearly values for any country did exist.
4. Curative (acute) care beds in hospitals per 100,000 population (beds). This variable was retrieved from Eurostat for 2010 - 2015 (original variable: Hospital beds by type of care (hlth_rs_bds). Data were available for all countries excluding the United Kingdom (UK). Data on acute care beds from the UK were retrieved from a different, but similarly recognized source (WHO Regional Office for Europe, 2019) for 2010 – 2013 (total curative care beds in hospitals, HP.1, Indicator code: hospBed.curative). Population values (demo_pjan) of the respective years were used to calculate the number of curative care beds in the UK per 100,000 population. The final score of beds was calculated as the mean of available yearly values, since yearly values for some countries were missing. This variable was included to represent a country’s healthcare infrastructure in the hospital sector. We wanted to determine whether beds potentially succeeded in significantly contributing to AMR variance explanation, where hsp had failed to do so. Therefore, we believed beds might be an important health system-dependent variable, which is specific for hospitals where the analyzed bacterial samples were obtained.
5. Total all causes disability-Adjusted Life Years (DALYs): DALY is an indicator on burden of disease and represents the lost years of healthy life. It is the sum of years lost due to premature death (YLL) and years lived with disability (YLD). DALY scores were given per 100 thousand population and means of the yearly values of 2010 – 2015 were calculated for each country (GBD Results Tool).
6. Average in-patient length of stay (ALOS) in days as the mean of 2000 - 2016, retrieved from OECD data (Dataset: Health Care Utilisation: Hospital average length of stay by diagnostic categories). Missing values were supplemented via Eurostat (hlth_co_inpst). Non-missing values were tested for concordance between both datasets (89%). In case of discordance, the values were retrieved from the OECD database.
7. Average hospital discharges (disc) per 1000 population as the mean of 2010 – 2016. Data of total cases were retrieved from Eurostat (all causes of diseases (A00-Z99) excluding V00-Y98, [hlth_co_disch1]) and OECD (Dataset: Health Care Utilisation: Hospital discharges by diagnostic categories, all causes). Missing values in OECD were supplemented via Eurostat (hlth_co_inpst). Both databases (Eurostat and OECD) were merged following the rule, that Eurostat data was included only when the information was missing in the OECD database (as of 5 June 2019). Non-missing values were tested for concordance between both datasets (72%). In case of discordance, the values were retrieved from the OECD database. Finally, the yearly values (2010 - 2015) were divided by the corresponding population (demo_pjan) and the 2010 - 2015 arithmetic mean comprised the final variable value.
8. Climatic variables: warm months warming (wmw = wm_warming), cold months warming (cmw = cm_warming), warm months temperature (wmt = wm_temp) and cold months temperature (cmt = cm_temp). The data were retrieved from the World Bank Climate Change Knowledge Portal (CCKP) and used in our previous work [9].
9. Since CRGN, FRGN and NRGN represent multi-species entities with various carbapenem-resistance probabilities; the species identity, and thus its isolation frequency of each species are expected to contribute to cumulative CRGN. Therefore, we calculated the proportion of each entity-specific isolate among all reported isolates (2013 - 2016). We thereby distinguished between *E. coli* (ECpr), *K. pneumoniae* (KPpr), *P. aeruginosa* (PApr) and *Acinetobacter* spp. (Acpr) proportions, which all sum up to 1. The proportions were calculated based on EARS-net tables for isolates tested against carbapenem resistance. This was achieved through dividing the total number of invasive entity specific isolates, e.g. for *E. coli* (Σ B_EC_ = B_2013_ + B_2014_ + B_2015_ + B_2016_)_EC_ by the total number of invasive isolates of all entities (Σ B_GN_ = B_2013_ + B_2014_ + B_2015_ + B_2016_)_EC+KP+PA+Ac_ with ECpr = Σ B_EC_/Σ B_GN_. KPpr, PApr and Acpr were calculated analogous to ECpr. We chose 2013 as starting year of observation for this variable due to the inconsistent reporting of *Acinetobacter* spp. before 2013.
10. Finally, we calculated the difference in proportion between each pair of the above mentioned proportion via subtracting one from the other (e.g. EADP = ECpr – Acpr; EKDP = ECpr – KPpr, etc.).

Table S1 provides further information on all variables used in this study, including raw data sources, accession codes and references.

**Multivariable analysis**

**Assumptions**

We used log-linear regression for multivariable analysis. Several assumptions must be met if we want to generalize conclusions drawn from a regression model based on a sample [10]. Briefly, these are:

1. no perfect multicollinearity (i.e. when a predictor is strongly related to at least one other predictor)
2. no autocorrelation (i.e. residuals for any two observations should be independent rather than correlated)
3. normally distributed errors
4. linear relationship between the outcome and all predictors
5. no heteroscedasticity
6. non-zero variance of predictors
7. independence of all outcome variable values
8. no underfitting (external variables which could influence the outcome by correlating with predictors) and overfitting (to many predictors)
9. variable type; in which predictors are quantitative or with two categories and outcome is quantitative, continuous and unbounded.

Assumption a) can be checked by calculating the variance inflation factor (VIF). A rule of thumb is that VIF should be less than 10, while an average VIF which is substantially larger than 1 might indicate some bias. To predict potential multicollinearity before running regression analysis, the strength of correlation between all independent variables was investigated using the Spearman correlation coefficient. Assumption b) can be tested by the Durbin-Watson test. A rule of thumb is that values less than 1 or greater than 3 are a source of concern.

Assumptions c) to e) on the other hand, were graphically checked, while assumption c) was additionally checked through various means, such as P-P diagrams or tested via the SW test in addition to graphical assessment.

Assumptions f) and g) are pre-defined and given by the collected data while h) is hardly to be perfectly assured. Yet, adjusting for variables which are potentially associated with the outcome (e.g. DDD, CPI etc.) in the model would decrease the probability of underfitting. Finally, most of the assumptions stated in i) are given with the exception of the property for an unbounded, continuous dependent variable, since the AMR (i.e. dependent) variables represent proportions with values theoretically between (and including) 0 and 1. It is worth to note that no variable in practice is completely unbounded and continuous [11].

Assumptions of regression models were finally examined in order to ascertain the validity of the obtained models. Information on assumption analysis is provided below. No major concerns for regression assumptions could be drawn from the analysis, although non-normal residues were observed but judged as no source of concern. (Supplementary Figures S1 – 4 and Supplementary Tables S5-12). All supplementary figures were prepared with IBM SPSS Statistics 26 – 27.

**Transformations and interpretations**

Fitting a classical linear regression model for data where the dependent variable is a proportion could lead to fitted values less than zero or greater than one. Hence, such a model is not appropriate for the data at hand (see above). Fitting a logistic regression model to the data requires data at the individual level (in contrast to the aggregated EARS-net data). As an alternative solution, so-called log-linear models were applied instead. For this purpose, the dependent variable was log-transformed by the natural logarithm function “ln(AMR)”, yielding a new variable (log_AMR) for each respective dependent variable (CRGN, FRGN and NRGN). For interpretation of these log-linear models, regression coefficients of each independent variable were re-transformed back to standard linear scale using the fact that logarithmic and exponential functions are inverses (ln(y) = log_e_(y) = x 🡪 y =e^x^). Only models M1.M, M3.M and M2.M (M3.M precursor) and analogous M1.S, M2.S and M3.S were validated and interpreted. Diagnostic models (M4.S – M17.S) were not validated and not interpreted. The interpretation of M18.S was compared to that of M3.M.

**Selection of independent factors**

For each dependent variable (M1.S, M2.S, M4.S and M5.S), we initially ran regression analysis by including all given suspected predictors and then eliminating non-significant predictor variables using stepwise forward selection of only independent, predictors (including interaction terms), i.e. those with significant regression coefficients (inclusion criterion F ≤ 0.05; exclusion criterion F ≥ 0.1) unless otherwise indicated. All determination coefficients are represented by the adjusted R^2^ of each model.

**Analysis of moderation**

Interaction terms were used as independent variables and calculated by multiplying the nurses variable with the main effect variable of interest, those were isolation frequency variables and variables of difference in proportion (points 12 and 13 ‘Potential confounders’ section). Analysis of moderation effects were performed as suggested by Baron and Kenny [12].

**Model equations**

Following CRGN models were obtained. Details on models M1.M, M2.M and M3.M can be taken from Table 2 (main text). Models M1.S, M2.S, M3.S, M4.S, M5.S, M6.S, M7.S, M8.S and M9.S are presented herein. Comparisons between model interpretations can be taken from Suppl. Table S4. Only independent variables with significant regression coefficients (p < 0.05) are considered in model equations 1 and 2. Ø denotes p > 0.05, * p < 0.05, ** p < 0.01 and *** p < 0.001.

1. log_CRGN = - 1.644* - 0.009 CPI*** + 0.029 DDD* + 8.48 Acpr*** - 0.080* nurses (M1.S)
2. log_CRGN = - 1.710** - 0.009 CPI*** + 0.033 DDD* + 6.556 Acpr*** - 0.125 intEAPD** (M2.S)
3. log_CRGN = - 1.684** - 0.010 CPI*** + 0.030 DDD* + 7.665 Acpr*** - 0.572 intEAlos** (M3.S)
4. log_FRGN = - 1.153** - 0.006 CPI*** + 0.022 DDD*** + 0.007 wmt*** + 0.040 cmw***

-0.023 wmw** (M4.S)

1. log_NRGN = - 2.801** - 0.006 CPI*** + 0.024 DDD** + 0.004 wmt* + 2.472 KPpr* + 3E-05 DALYs* (M5.S)
2. log_FRGN = - 0.062^Ø^ - 0.007 CPI*** + 0.010 DDD* + 0.008 log_Acpr^Ø^ - 0.015 intEAlos^Ø^ (M6.S)
3. log_NRGN = - 0.764^Ø^ - 0.011 CPI*** + 0.011 DDD^Ø^ + 0.093 log_Acpr^Ø^ - 0.172 intEAlos^Ø^ (M7.S)
4. log_MRSA = - 1.280^Ø^ - 0.013 CPI*** + 0.089 DDD*** - 0.253 log_Acpr^Ø^ - 0.279 intEAlos^Ø^ (M8.S)
5. log_CRGN = - 1.115^Ø^ - 0.008 CPI*** + 0.036 DDD** + 0.454 log_Acpr*** - 14.898 intEAdisc** (M9.S)
6. log_FRGN = - 0.247^Ø^ - 0.008 CPI*** + 0.019 DDD* + 0.021 log_Acpr^Ø^ + 3.591 intEAdisc^Ø^ (M10.S)
7. log_NRGN = 0.945* - 0.011 CPI*** + 0.009 DDD^Ø^ + 0.123 log_Acpr^Ø^ + 6.878 intEAdisc^Ø^ (M11.S)
8. log_CRGN = - 0.312^Ø^ - 0.008 CPI*** + 0.038 DDD** + 0.439 log_Acpr*** - 2716.629 intEADALYs** (M12.S)
9. log_FRGN = 0.207^Ø^ - 0.008 CPI*** + 0.019 DDD* + 0.012 log_Acpr^Ø^ + 424.741 intEADALYs^Ø^ (M13.S)
10. log_NRGN = 0.887* - 0.011 CPI*** + 0.010 DDD^Ø^ + 0.109 log_Acpr^Ø^ + 866.490 intEADALYs^Ø^ (M14.S)
11. log_CRPA = 0.009^Ø^ - 0.008 CPI*** + 0.039 wmw* (M15.S = M4.0 in [9])
12. log_CRKP = - 6.387^***^ - 0.011 CPI** + 0.103 DDD** + 0.026 wmt* (M16.S = M3.0 in [9])
13. log_CRAc = 2.567** - 0.012 CPI** - 0.175* nurses (M17.S)
14. log_CRGN = 0.045^Ø^ - 0.008 CPI*** + 0.029 DDD* + 0.490 log_Acpr*** - 0.569 int_alt_EALOS** (M18.S)
15. log_CRGN = - 1.008^Ø^ - 0.012 CPI*** + 0.032 DDD* + 6.513 Acpr** - 0.826 intEAlos_H* (M19.S)

Variables not included by the model (p ≥ 0.05):

M1.S hsp, DALYs, wmt, cmt, wmw, cmw, docs, ac_beds, ALOS, ECpr, KPpr, PApr

M2.S nurses, EKPD, EPPD, EAPD, intEKPD, intEPPD

**Estimation of AMR proportions for European countries not covered by the EARS-net surveillance program**

Model M3.M was used to estimate CRGN proportions for countries outside the EU/EAA region. The selected countries were intended to represent different geographic regions within Europe, including countries located in the geographical periphery of the continent or transcontinental countries as reported before [9]. Thus, Belarus, Serbia, Switzerland and Turkey were selected, which were characterized by the availability of empirical AMR data collected by the WHO Office for the European Region in the Central Asian and Eastern European Surveillance of Antimicrobial Resistance (CAESAR) program [13], allowing comparison to the estimations made by the different models. Nurse-density data could not be obtained from reliable sources in English or German language for Belarus and Serbia, which restricted the M3.M estimation of CRGN prevalence on Switzerland and Turkey.

Values of CPI variables for all four countries could be obtained from the same sources as for the EU/EEA countries analyzed in this study (Transparency International). Antibiotic (J01) consumption volumes were obtained for Turkey [14] and Switzerland [15] from different sources.

The estimated CRGN proportions were then compared to cumulative prevalence calculated from published empirical data within the CAESAR program. This surveillance program includes countries within the WHO European Region that do not participate in EARS-net. In the published CAESAR documents, the total number of bacterial isolates (B) and percentage of resistant isolates (AMR_y_) for a given year are shown. We used these figures to estimate the absolute number of resistant isolates (R) by multiplying B with AMR_y_ for a given entity. We then calculated the cumulative CRGN prevalence for the reported years (2013 - 2016 for Switzerland and Turkey) by dividing ∑ R by ∑ B in analogy to EQ1 in our previous work [9].

**Allocation of countries to either sub-geographic group (variable geo_2G)**

The 30 EU/EEA countries were divided into two geopolitical groups primarily based on their geographic localization and secondarily based on their historical political systems. One group comprised the Northern and Western European countries (NWC), while the other contained countries from the south and east of Europe (SEC). For central European countries, the secondary criterion was being (part of) a former socialist country during the Cold War era. If true, these countries (e.g. Hungary, Czechia, Slovakia and Slovenia) were assigned to SEC group, while Germany and Austria were assigned to the NWC group. Baltic countries (Estonia, Latvia and Lithuania) were allocated to the NWC group due to their geographic localization, which is the primary criterion. The affiliation of each country to one of the two groups can be taken from Figure 1 and Supplementary Table 2 (variable geo_2G).

**Comparison between sub-geographic groups**

The Mann-Whitney-Test (MW) was run to compare metric variables in both groups. The MW test is a non-parametric test that works by ranking the data, similar to Spearman’s correlation coefficient. This test represents higher scores by larger ranks and lower scores by smaller ranks. A statistical test investigates whether the mean ranks of the two groups are different, i.e.; whether the two groups derive from two subpopulations. This test was favored over the t-test due to the relatively small size of our sample; as non-parametric tests are usually applied when the assumptions for parametric tests, such as the t-test are not met.

**Other Software**

https://mapchart.net/ was used to create the map of European countries shown in Figure 1. Zotero was used for reference style & management.

**ECDC mandatory disclaimer**

"The views and opinions of the authors expressed herein do not necessarily state or reflect those of the ECDC. The accuracy of the authors' statistical analysis and the findings they report are not the responsibility of ECDC. ECDC is not responsible for conclusions or opinions drawn from the data provided. ECDC is not responsible for the correctness of the data and for data management, data merging and data collation after provision of the data. ECDC shall not be held liable for improper or incorrect use of the data".

**Supplementary Figures**

**Supplementary Figure S1a. M1.S. Assumption (c), normally distributed errors. Left: histogram of standardized residuals. Right: P-P diagram. Test statistics of ZRE_log_CRGN, Kolmogorov-Smirnov p = 0.200, Shapiro-Wilk p = 0.981.**

**
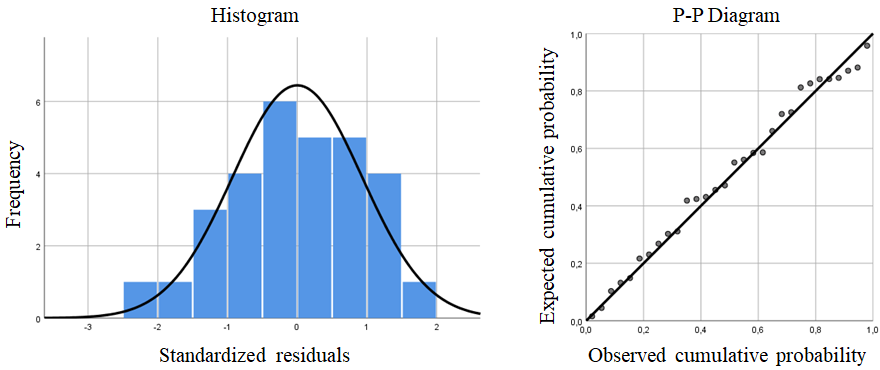
**

**Supplementary Figure S1b. M1.S. Assumption (d). Linear relationship between the dependent variable log_CRGN and independent predictors.**

**Supplementary Figure S1c. M1.S. Assumption (e). Partial regression diagrams for the assessment of heteroscedasticity.**

**Supplementary Figure S2a. M1.M. Assumption (c), normally distributed errors. Left: histogram of standardized residuals. Right: P-P diagram. Test statistics of ZRE_log_CRGN, Kolmogorov-Smirnov p = 0.136, Shapiro-Wilk p = 0.018.**


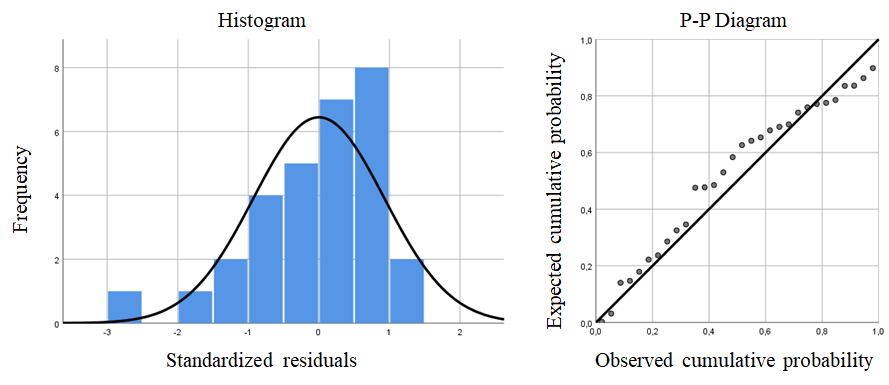


**Supplementary Figure S2b. M1.M. Assumption (d). Linear relationship between the dependent variable log_CRGN and independent predictors (log_Acpr; consult Suppl. Fig. S1b for other predictors).**

**Supplementary Figure S2c. M1.M. Assumption (e). Partial regression diagrams for the assessment of heteroscedasticity.**

**Supplementary Figure S3a. M2.S. Assumption (c), normally distributed errors. Left: histogram of standardized residuals. Right: P-P diagram. Test statistics of ZRE_log_CRGN, Kolmogorov-Smirnov p = 0.200, Shapiro-Wilk p = 0.704.**

**
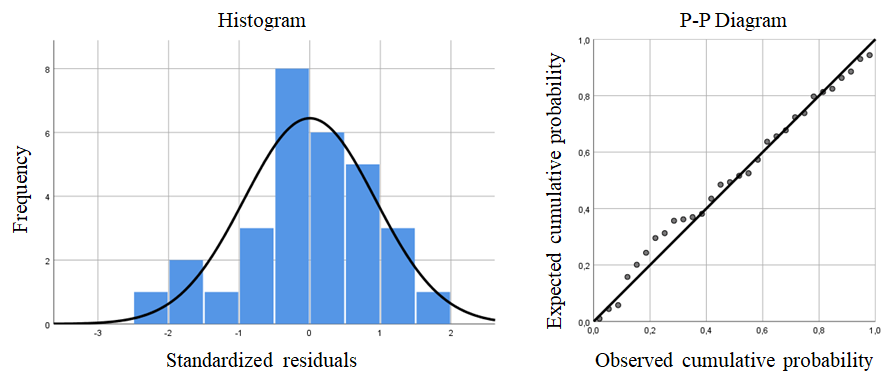
**

**Supplementary Figure S3b. M2.S. Assumption (d). Linear relationship between the dependent variable log_CRGN and independent predictors (intEAPD; consult Suppl. Figs. S1b and S1e for other predictors).**

**Supplementary Figure S3c. M2.S. Assumption (e). Partial regression diagrams for the assessment of heteroscedasticity.**

**Supplementary Figure S4a. M2.M. Assumption (c), normally distributed errors. Left: histogram of standardized residuals. Right: P-P diagram. Test statistics of ZRE_log_CRGN, Kolmogorov-Smirnov p = 0.184, Shapiro-Wilk p = 0.017.**

**
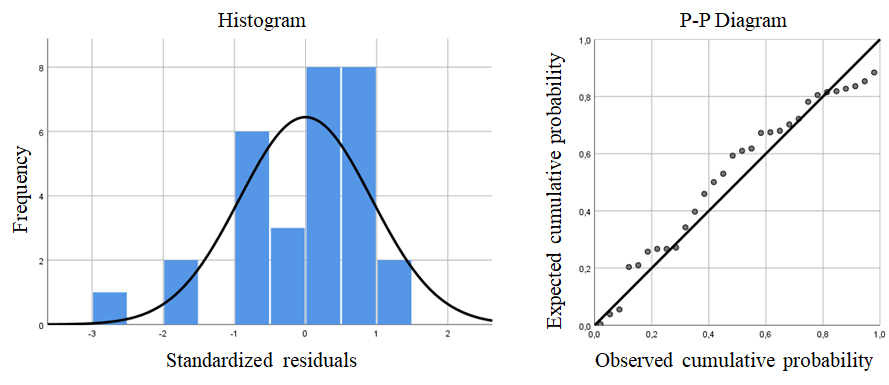
**

**Supplementary Figure S4b. M2.M. Assumption (e). Partial regression diagrams for the assessment of heteroscedasticity.**

**Supplementary Figure S5a. M3.S. Assumption (c), normally distributed errors. Left: histogram of standardized residuals. Right: P-P diagram. Test statistics of ZRE_log_CRGN, Kolmogorov-Smirnov p = 0.200, Shapiro-Wilk p = 0.278.**

**
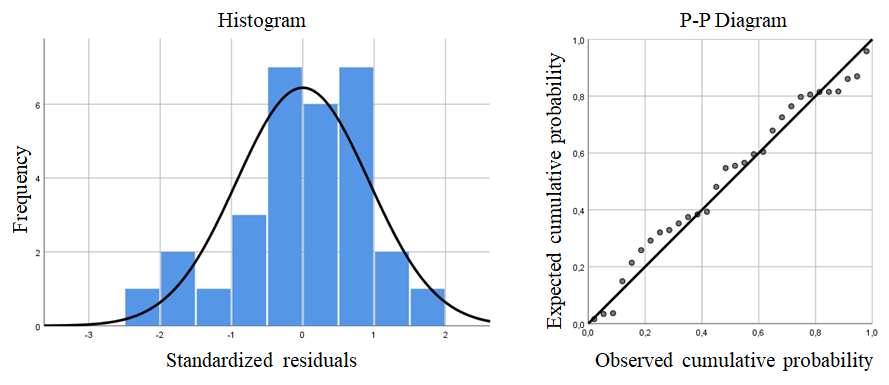
**

**Supplementary Figure S5b. M3.S. Assumption (d). Linear relationship between the dependent variable log_CRGN and independent predictors (intEAlos; consult Suppl. Figs. S1b and S2b for other predictors).**

**Supplementary Figure S5c. M3.S. Assumption (e). Partial regression diagrams for the assessment of heteroscedasticity.**

**
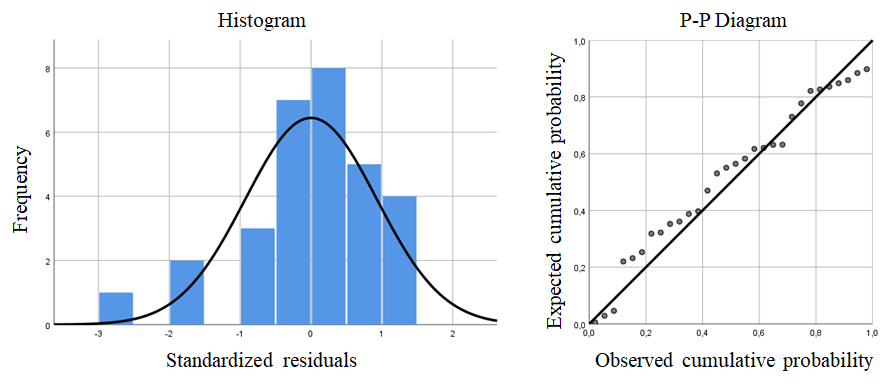
Supplementary Figure S6a. M3.M. Assumption (c), normally distributed errors. Left: histogram of standardized residuals. Right: P-P diagram. Test statistics of ZRE_log_CRGN, Kolmogorov-Smirnov p = 0.926, Shapiro-Wilk p = 0.039.**

**Supplementary Figure S6b. M3.M. Assumption (e). Partial regression diagrams for the assessment of heteroscedasticity.**

**Supplementary Figure S7a. M4.M. Assumption (c), normally distributed errors. Left: histogram of standardized residuals. Right: P-P diagram. Test statistics of ZRE_log_CRGN, Kolmogorov-Smirnov p = 0.200, Shapiro-Wilk p = 0.171.**


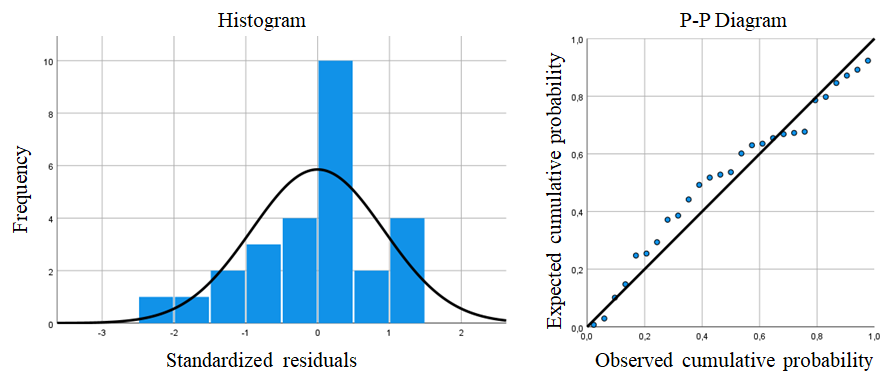


**Supplementary Figure S6b. M3.S. Assumption (d). Linear relationship between the dependent variable log_CRGN and independent predictors (intEAlos_H; consult Suppl. Figs. S1b and S2b for other predictors)**

**Supplementary Figure S7c. M4.M. Assumption (e). Partial regression diagrams for the assessment of heteroscedasticity.**

**Supplementary Tables**

**Supplementary Table 1. Description of the variables used in statistical analysis.**

| **Variable** | **Description** | **Created** | **Raw data source** |
| --- | --- | --- | --- |
| CRGN | Cumulative proportion (%) of *K. pneumoniae* + *P. aeruginosa* + *Acinetobacter* spp. + *E. coli* isolates resistant to carbapenems (2011-2016), log-transformed (ln) for modeling. | This study | ECDC EARS-net [1] |
| FRGN | Cumulative proportion (%) of *K. pneumoniae* + *P. aeruginosa* + *Acinetobacter* spp. + *E. coli* isolates resistant to fluoroquinolones (2011-2016), log-transformed (ln) for modeling. | This study |  |
| NRGN | Cumulative proportion (%) of *K. pneumoniae* + *P. aeruginosa* + *Acinetobacter* spp. + *E. coli* isolates resistant to aminoglycosides (2011-2016), log-transformed (ln) for modeling. | This study |  |
| ECpr | Proportion of reported *E. coli* isolates among all reported gram-negative isolates (2013-2016). | This study |  |
| KPpr | Proportion of reported *K. pneumoniae* isolates among all reported gram-negative isolates (2013-2016). | This study |  |
| PApr | Proportion of reported *P. aeruginosa* isolates among all reported gram-negative isolates (2013-2016). | This study |  |
| Acpr | Proportion of reported *Acinetobacter* spp. isolates among all reported gram-negative isolates (2013-2016), log-transformed (ln) for modeling. | This study |  |
| EKPD | Difference in proportions of reported *E. coli* isolates and reported *K. pneumoniae* among all reported gram-negative isolates (2013-2016), i.e. ECpr – KPpr. | This study |  |
| EPPD | Difference in proportions of reported *E. coli* isolates and reported *P. aeruginosa* among all reported gram-negative isolates (2013-2016), i.e. ECpr – PApr. | This study |  |
| EAPD | Difference in proportions of reported *E. coli* isolates and reported *Acinetobacter* spp. among all reported gram-negative isolates (2013-2016), i.e. ECpr – Acpr. | This study |  |
| KAPD | Difference in proportions of reported *K. pneumoniae* isolates and reported *Acinetobacter* spp. among all reported gram-negative isolates (2013-2016), i.e. KPpr – Acpr. | This study |  |
| PAPD | Difference in proportions of reported *P. aeruginosa* isolates and reported *Acinetobacter* spp. among all reported gram-negative isolates (2013-2016), i.e. PApr – Acpr. | This study |  |
| KPPD | Difference in proportions of reported *K. pneumoniae* isolates and reported *P. aeruginosa* among all reported gram-negative isolates (2013-2016), i.e. KPpr – PApr. | This study |  |
| CRAc | *Acinetobacter* spp. isolates resistant to carbapenems (2012-2016), log-transformed (ln) for modeling. | This study |  |
| CRKP | *K. pneumoniae* isolates resistant to carbapenems (2011-2016), log-transformed (ln) for modeling. | Kaba et al. [9] |  |
| CRPA | *P. aeruginosa* isolates resistant to carbapenems (2011-2016), log-transformed (ln) for modeling. |  |  |
| MRSA | MRSA proportion (%) (*Staphylococcus aureus* isolates (2011-2016), log-transformed (ln) for modeling. |  |  |
| DDD | Total consumption of antibacterials for systemic use (ATC group J01) in defined daily doses per day and 1000 population (primary care sector; mean of 2010-2015) |  | ECDC ESAC [16] |
| nurses | Nurse-density, using [hlth_rs_prsns] or [Nurses (indicator). doi: 10.1787/283e64de-en] per 1000 population [demo_pjan], mean of 2010-2015. | This study | Eurostat [17,18] OECD [6] |
| docs | Physician density [hlth_rs_phys] per 1000 population [demo_pjan] , mean of 2010-2015. | Kaba et al. [7] | Eurostat [18,19] |
| hsp | Health spending (hsp) as % of GDP, [SH.XPD.CHEX.GD.ZS]. |  | World Bank [20] |
| beds | Mean of curative (acute) care beds in hospitals per 100,000 population (2010-2015). Hospital beds by type of care [hlth_rs_bds] and Total number of hospital beds (HP.1). |  | Eurostat [21], WHO [22] |
| DALYs | Disability-Adjusted Life Years, proportion per 100 thousand population, mean of 2010 – 2015. | This study | GBD [23] |
| ALOS | Hospital average length of stay (all causes), [hlth_co_inpst]. | This study | Eurostat [24], OECD [25] |
| disc | Hospital discharges (all causes) per 1000 population, mean of 2010-2015. | This study | OECD [26], Eurostat [18, 27] |
| CPI | Corruption Perceptions Index score, sum of points 2012-2015. | Kaba et al. [9] | Transparency Int. [28] |
| wmt | Sum of temperature means [°C] (1991-2015) for the six warmest months (May, June, July, August, September and October) | Kaba et al. [9] as wm_temp | World Bank CCKP [29] |
| cmt | Sum of temperature means [°C] (1991-2015) for the six coldest months (November, December, January, February, March and April) | Kaba et al. [9] as cm_temp |  |
| wmw | Net year-wise increase/ decrease in mean monthly temperatures [°C] (1991-2015) for May, June, July, August, September and October | Kaba et al. [9] as wm_net_warming |  |
| cmw | Net year-wise increase/ decrease in mean monthly temperatures [°C] (1991-2015) for November, December, January, February, March and April | Kaba et al. [7] as cm_net_warming |  |
| nurses_H | Density of nursing professionals and midwives employed in hospital [HLTH_RS_PRSHP1] per 1000 population, mean of 2011-2015. | This study | Eurostat [18,30] |
| alt_nurses | Alternative calculation of nurse-density, using [hlth_rs_prsns] or [Nurses (indicator). doi: 10.1787/283e64de-en] per 1000 population [demo_pjan], mean of 2010-2015. Consult page 2, point 3 of this document for details. | This study | Eurostat [17,18] OECD [6] |
| intEKPD | Interaction term nurses * EKPD | This study | See above |
| intEPPD | Interaction term nurses * EPPD | This study |  |
| intEAPD | Interaction term nurses * EAPD | This study |  |
| intEAPD_H | Interaction term nurses_H * EAPD | This study |  |
| int_alt_EAPD | Interaction term alt_nurses * EAPD | This study |  |
| intEAlos | intEAPD/ALOS | This study |  |
| intEAlos_H | intEAPD_H/ALOS | This study |  |
| int_alt_EAlos | Int_alt_EAPD/ALOS | This study |  |
| intEAdisc | intEAPD/disc | This study |  |
| intEADALYs | intEAPD/DALYs | This study |  |
| geo_2G | Binary variable, representing the geographic allocation of each country into either the “NWC” or the “SEC” groups | Kaba et al. [9] | --- |
| nurses_2G | Binary variable, representing whether the calculated nurse-density of a given country is lower than the EU/EEA nurses median (true; false) | This study | See above |
| docs_2G | Binary variable, representing whether the calculated physician density of a given country is lower than the EU/EEA docs median (true; false) | This study | See above |

**Supplementary Table S2. Values of basic isolate related variables and geographical affiliation. geo_2G indicates the country’s group affiliation, either to the Northern/ Western (NWC = 1) or to the Southern/ Eastern (SEC = 2) country group. Transformed variables were calculated as indicated above based on the basic isolate related variables.**

| **ID** | **Country** | **geo_2G** | **CRGN** | **FRGN** | **NRGN** | **ECpr** | **KPpr** | **PApr** | **Acpr** | **CRAc** |
| --- | --- | --- | --- | --- | --- | --- | --- | --- | --- | --- |
| 1 | Austria | 1 | 0.016 | 0.185 | 0.070 | 0.728 | 0.159 | 0.102 | 0.011 | 0.091 |
| 2 | Belgium | 1 | 0.010 | 0.226 | 0.085 | 0.782 | 0.124 | 0.087 | 0.006 | 0.018 |
| 3 | Bulgaria | 2 | 0.166 | 0.456 | 0.435 | 0.399 | 0.268 | 0.112 | 0.221 | 0.665 |
| 4 | Croatia | 2 | 0.106 | 0.321 | 0.251 | 0.579 | 0.194 | 0.136 | 0.091 | 0.902 |
| 5 | Cyprus | 2 | 0.146 | 0.404 | 0.242 | 0.471 | 0.229 | 0.157 | 0.143 | 0.731 |
| 6 | Czechia | 2 | 0.020 | 0.314 | 0.225 | 0.491 | 0.344 | 0.145 | 0.020 | 0.045 |
| 7 | Denmark | 1 | 0.004 | 0.111 | 0.060 | 0.742 | 0.165 | 0.081 | 0.012 | 0.034 |
| 8 | Estonia | 1 | 0.013 | 0.154 | 0.094 | 0.719 | 0.207 | 0.068 | 0.006 | 0.445 |
| 9 | Finland | 1 | 0.005 | 0.104 | 0.047 | 0.808 | 0.122 | 0.063 | 0.007 | 0.014 |
| 10 | France | 1 | 0.025 | 0.193 | 0.116 | 0.694 | 0.149 | 0.129 | 0.029 | 0.050 |
| 11 | Germany | 1 | 0.013 | 0.186 | 0.071 | 0.771 | 0.131 | 0.075 | 0.024 | 0.061 |
| 12 | Greece | 2 | 0.470 | 0.561 | 0.455 | 0.305 | 0.291 | 0.184 | 0.220 | 0.918 |
| 13 | Hungary | 2 | 0.129 | 0.342 | 0.256 | 0.480 | 0.182 | 0.209 | 0.128 | 0.512 |
| 14 | Iceland | 1 | 0.045 | 0.088 | 0.034 | 0.205 | 0.036 | 0.614 | 0.145 | 0.000 |
| 15 | Ireland | 1 | 0.006 | 0.212 | 0.110 | 0.803 | 0.113 | 0.061 | 0.023 | 0.026 |
| 16 | Italy | 2 | 0.152 | 0.460 | 0.272 | 0.595 | 0.217 | 0.117 | 0.071 | 0.812 |
| 17 | Latvia | 1 | 0.088 | 0.339 | 0.268 | 0.526 | 0.287 | 0.050 | 0.136 | 0.733 |
| 18 | Lithuania | 1 | 0.053 | 0.280 | 0.233 | 0.665 | 0.222 | 0.051 | 0.063 | 0.779 |
| 19 | Luxembourg | 1 | 0.008 | 0.253 | 0.103 | 0.777 | 0.140 | 0.071 | 0.012 | 0.000 |
| 20 | Malta | 2 | 0.028 | 0.300 | 0.141 | 0.674 | 0.223 | 0.079 | 0.024 | 0.133 |
| 21 | Netherlands | 1 | 0.003 | 0.122 | 0.062 | 0.797 | 0.124 | 0.068 | 0.011 | 0.021 |
| 22 | Norway | 1 | 0.004 | 0.093 | 0.050 | 0.772 | 0.167 | 0.053 | 0.008 | 0.025 |
| 23 | Poland | 2 | 0.081 | 0.425 | 0.284 | 0.563 | 0.245 | 0.097 | 0.095 | 0.567 |
| 24 | Portugal | 2 | 0.055 | 0.320 | 0.194 | 0.605 | 0.227 | 0.136 | 0.032 | 0.609 |
| 25 | Romania | 2 | 0.289 | 0.514 | 0.451 | 0.403 | 0.314 | 0.106 | 0.177 | 0.831 |
| 26 | Slovakia | 2 | 0.096 | 0.518 | 0.383 | 0.488 | 0.273 | 0.148 | 0.091 | 0.341 |
| 27 | Slovenia | 2 | 0.025 | 0.238 | 0.121 | 0.757 | 0.143 | 0.077 | 0.022 | 0.337 |
| 28 | Spain | 2 | 0.028 | 0.305 | 0.153 | 0.725 | 0.165 | 0.099 | 0.011 | 0.642 |
| 29 | Sweden | 1 | 0.005 | 0.104 | 0.054 | 0.794 | 0.144 | 0.055 | 0.007 | 0.033 |
| 30 | United Kingdom | 1 | 0.005 | 0.144 | 0.084 | 0.773 | 0.135 | 0.073 | 0.019 | 0.016 |
| 31 | Switzerland |  |  |  |  | 0.771 |  |  | 0.011 |  |
| 32 | Turkey |  |  |  |  | 0.400 |  |  | 0.200 |  |

**Supplementary Table S3a. Values of the potential predictor and confounding variables.**

| **ID** | **Country** | **DDD** | **docs** | **nurses** | **nurses_H** | **hsp** | **CPI** | **wmt** | **cmt** | **wmw** | **cmw** | **beds** | **ALOS** | **DALYs** | **disc** |
| --- | --- | --- | --- | --- | --- | --- | --- | --- | --- | --- | --- | --- | --- | --- | --- |
| 1 | Austria | 14.60 | 4.96 | 6.70 | 6.90 | 60.90 | 286 | 79.4 | 6.9 | 10.8 | 13.9 | 593 | 8.18 | 26262 | 268.4 |
| 2 | Belgium | 28.25 | 2.96 | 10.19 | 7.43 | 61.40 | 303 | 92.4 | 34.1 | 3.5 | 12.6 | 530 | 7.13 | 27245 | 170.3 |
| 3 | Bulgaria | 19.78 | 4.00 | 4.36 | 3.21 | 46.40 | 166 | 110.3 | 24.4 | 9.0 | 13.2 | 572 | 7.42 | 38847 | 291.4 |
| 4 | Croatia | 20.92 | 3.02 | 1.09 | 0.82 | 45.90 | 193 | 106.5 | 30.6 | 12.5 | 11.5 | 386 | 10.46 | 33818 | 158.2 |
| 5 | Cyprus | 29.68 | 3.48 | 4.72 | 4.39 | 40.00 | 253 | 150.4 | 82.0 | 8.0 | 5.8 | 346 | 5.80 | 23590 | 78.3 |
| 6 | Czechia | 18.53 | 3.65 | 8.02 |  | 43.70 | 204 | 90.2 | 14.7 | 10.7 | 16.4 | 440 | 10.24 | 30757 | 203.8 |
| 7 | Denmark | 16.45 | 3.65 | 9.83 | 7.21 | 61.60 | 364 | 83.7 | 20.4 | 0.3 | 10.5 | 292 | 6.43 | 27225 | 154.0 |
| 8 | Estonia | 11.73 | 4.60 | 5.98 | 4.49 | 36.70 | 271 | 79.0 | -4.4 | 3.4 | 11.9 | 374 | 7.71 | 34001 | 171.3 |
| 9 | Finland | 18.62 | 3.31 | 9.76 | 1.08 | 55.50 | 358 | 61.0 | -33.4 | 3.8 | 13.4 | 320 | 11.64 | 27679 | 175.9 |
| 10 | France | 29.27 | 3.33 | 9.20 | 5.71 | 65.40 | 281 | 98.5 | 45.6 | 6.4 | 7.4 | 335 | 5.92 | 25035 | 167.2 |
| 11 | Germany | 14.72 | 3.98 | 10.36 | 5.34 | 65.80 | 317 | 90.9 | 24.2 | 4.4 | 11.3 | 620 | 9.66 | 29298 | 250.8 |
| 12 | Greece | 35.25 | 6.26 | 1.83 | 2.07 | 52.10 | 165 | 123.1 | 48.7 | 11.0 | 7.3 | 360 | 7.43 | 27866 | 197.7 |
| 13 | Hungary | 15.88 | 3.09 | 4.86 | 2.88 | 44.30 | 214 | 106.2 | 24.4 | 12.4 | 14.5 | 434 | 9.21 | 36512 | 203.0 |
| 14 | Iceland | 21.30 | 3.63 | 9.04 | 5.87 | 52.20 | 318 | 36.2 | -8.3 | -5.1 | -1.8 | 271 | 5.73 | 21099 | 122.8 |
| 15 | Ireland | 22.98 | 3.72 | 12.74 | 5.01 | 58.60 | 290 | 76.6 | 38.4 | -3.3 | 3.9 | 247 | 6.09 | 22190 | 133.6 |
| 16 | Italy | 27.92 | 4.70 | 5.16 | 4.47 | 53.70 | 172 | 109.4 | 41.5 | 9.2 | 9.4 | 281 | 7.58 | 26534 | 127.2 |
| 17 | Latvia | 12.83 | 3.15 | 4.87 | 3.27 | 45.30 | 213 | 81.8 | -0.9 | 3.7 | 11.4 | 349 | 7.75 | 39619 | 163.1 |
| 18 | Lithuania | 17.35 | 4.48 | 7.50 | 5.04 | 38.40 | 228 | 84.7 | 2.4 | 3.4 | 12.5 | 613 | 7.68 | 39351 | 235.4 |
| 19 | Luxembourg | 27.15 | 2.86 | 11.81 | 7.15 | 38.60 | 327 | 92.0 | 29.8 | 4.1 | 12.3 | 409 | 8.55 | 23356 | 145.7 |
| 20 | Malta | 22.72 | 3.40 | 7.12 | 6.13 | 53.30 | 228 | 144.3 | 87.5 | 8.1 | 2.8 | 273 | 7.68 | 25028 | 138.8 |
| 21 | Netherlands | 11.00 | 3.27 | 12.01 | 4.68 | 64.30 | 334 | 90.0 | 33.8 | 3.2 | 12.8 | 319 | 6.19 | 25245 | 109.6 |
| 22 | Norway | 16.18 | 4.30 | 16.76 | 9.03 | 54.70 | 345 | 48.1 | -27.4 | 3.8 | 8.0 | 356 | 5.34 | 23496 | 171.6 |
| 23 | Poland | 23.12 | 2.25 | 5.25 | 3.55 | 37.80 | 242 | 90.7 | 12.3 | 6.7 | 15.3 | 495 | 7.52 | 31212 | 166.2 |
| 24 | Portugal | 21.58 | 4.20 | 6.00 | 3.55 | 55.80 | 252 | 118.2 | 65.6 | -0.1 | 6.0 | 329 | 7.25 | 28032 | 85.7 |
| 25 | Romania | 31.48 | 2.62 | 0.45 | 0.39 | 30.20 | 176 | 100.9 | 14.8 | 13.6 | 14.8 | 491 | 7.94 | 36774 | 223.6 |
| 26 | Slovakia | 22.56 | 3.39 | 5.84 |  | 44.20 | 194 | 90.4 | 10.6 | 11.6 | 14.5 | 505 | 8.12 | 30579 | 190.5 |
| 27 | Slovenia | 14.38 | 2.62 | 2.31 | 1.42 | 51.70 | 236 | 95.1 | 21.2 | 11.4 | 11.8 | 428 | 7.13 | 28753 | 178.0 |
| 28 | Spain | 20.83 | 3.81 | 5.20 | 3.19 | 54.50 | 242 | 115.7 | 51.2 | 5.7 | 8.1 | 241 | 7.12 | 23757 | 100.6 |
| 29 | Sweden | 13.48 | 4.10 | 11.11 |  | 63.40 | 353 | 57.0 | -27.6 | 2.9 | 9.1 | 241 | 6.42 | 25637 | 159.3 |
| 30 | United Kingdom | 19.85 | 2.70 | 6.53 | 6.32 | 55.00 | 309 | 77.0 | 32.8 | -1.2 | 7.7 | 234 | 7.90 | 26202 | 130.3 |
| 31 | Switzerland | 5.83 |  | 10.91 |  |  | 343 | 76.3 | 12.0 | 4.5 | 5.2 | 371 | 9.53 |  |  |
| 32 | Turkey | 42.30 |  | 1.78 |  |  | 186 | 113.4 | 26.2 | 6.3 | 5.7 | 265 | 4.71 |  |  |

**Supplementary Table S3b. Practicing nursing professionals per 1000 population (HLTH_RS_PRSNS, Eurostat [17]).**

| **Country** | **2010** | **2011** | **2012** | **2013** | **2014** | **2015** |  |
| --- | --- | --- | --- | --- | --- | --- | --- |
| Austria | 6.54 | 6.64 | 6.67 | 6.71 | 6.82 | 6.84 | only nursing professionals working in hospitals |
| Belgium |  |  |  |  |  |  |  |
| Bulgaria | 4.28 | 4.29 | 4.38 | 4.46 | 4.40 | 4.36 |  |
| Croatia | 0.91 | 0.95 | 1.03 | 1.11 | 1.20 | 1.35 |  |
| Cyprus | 4.52 | 4.68 | 4.56 | 4.64 | 4.81 | 5.10 |  |
| Czechia |  |  |  |  |  |  |  |
| Denmark | 9.74 | 9.78 | 9.88 | 9.87 | 9.86 | 9.85 |  |
| Estonia | 6.12 | 6.23 | 6.16 | 5.63 | 5.74 | 6.01 |  |
| Finland | 9.17 | 9.40 | 9.75 | 10.25 | 10.25 |  |  |
| France |  |  |  |  |  |  |  |
| Germany | 9.84 | 10.13 | 10.18 | 10.53 | 10.72 | 10.78 |  |
| Greece | 1.84 | 1.87 | 1.82 | 1.82 | 1.81 | 1.82 | only nursing professionals working in hospitals |
| Hungary | 4.72 | 4.90 | 4.85 | 4.87 | 4.92 | 4.92 |  |
| Iceland | 8.35 | 8.68 | 9.10 | 9.40 | 9.31 | 9.39 |  |
| Ireland |  |  |  |  |  |  |  |
| Italy | 4.84 | 5.06 | 5.19 | 5.13 | 5.28 | 5.44 |  |
| Latvia |  |  |  |  |  |  |  |
| Lithuania | 7.27 | 7.47 | 7.55 | 7.51 | 7.57 | 7.62 |  |
| Luxembourg | 11.16 | 11.42 | 12.06 | 12.07 | 12.11 | 12.05 |  |
| Malta | 6.49 | 6.71 | 6.72 | 7.04 | 7.95 | 7.80 |  |
| Netherlands |  |  |  |  |  |  |  |
| Norway | 16.23 | 16.51 | 16.63 | 16.76 | 16.99 | 17.41 |  |
| Poland | 5.28 | 5.28 |  | 5.27 | 5.24 | 5.19 |  |
| Portugal |  |  |  |  |  |  |  |
| Romania | 0.27 | 0.32 | 0.44 | 0.51 | 0.55 | 0.61 |  |
| Slovakia |  |  |  |  |  |  |  |
| Slovenia | 2.12 | 2.19 | 2.21 | 2.33 | 2.44 | 2.59 |  |
| Spain | 5.16 | 5.23 | 5.24 | 5.13 | 5.15 | 5.29 |  |
| Sweden | 11.07 | 11.09 | 11.11 | 11.14 | 11.12 | 11.12 |  |
| United Kingdom | 6.71 | 6.62 | 6.44 | 6.46 | 6.49 | 6.48 | only nursing professionals working in hospitals |

**Supplementary Table S3c. Practising/ professionally active nurses per 1000 population (OECD [6]). Colored rows correspond to values merged with Eurostat data of Suppl. Table S8.**

| **Country** | **2010** | **2011** | **2012** | **2013** | **2014** | **2015** |  |
| --- | --- | --- | --- | --- | --- | --- | --- |
| Austria | 7.70 | 7.78 | 7.83 | 7.87 | 8.00 | 8.04 |  |
| Belgium | 9.59 | 9.81 | 10.02 | 10.30 | 10.58 | 10.83 |  |
| Bulgaria |  |  |  |  |  |  |  |
| Croatia |  |  |  |  |  |  |  |
| Cyprus |  |  |  |  |  |  |  |
| Czechia | 8.10 | 8.03 | 8.06 | 7.99 | 7.93 | 8.01 |  |
| Denmark | 15.83 | 16.01 | 16.31 | 16.52 | 16.70 | 16.90 |  |
| Estonia | 6.12 | 6.24 | 6.17 | 5.64 | 5.74 | 6.01 |  |
| Finland | 13.86 | 14.08 | 14.20 | 14.18 | 14.26 |  |  |
| France | 8.45 | 8.71 | 9.10 | 9.38 | 9.66 | 9.92 | professionally active |
| Germany | 11.53 | 11.87 | 11.96 | 12.34 | 12.55 | 12.65 |  |
| Greece | 3.45 | 3.47 | 3.34 | 3.29 | 3.23 | 3.21 |  |
| Hungary | 6.21 | 6.21 | 6.32 | 6.43 | 6.41 | 6.47 |  |
| Iceland | 14.54 | 14.82 | 15.16 | 15.45 | 15.33 | 15.45 |  |
| Ireland | 13.45 | 12.94 | 12.99 | 12.55 | 12.18 | 12.35 | professionally active |
| Italy |  | 6.48 | 6.41 | 6.14 | 6.15 | 6.11 |  |
| Latvia | 5.01 | 4.96 | 4.86 | 4.88 | 4.82 | 4.68 |  |
| Lithuania | 7.37 | 7.54 | 7.59 | 7.55 | 7.60 | 7.66 |  |
| Luxembourg | 11.05 | 11.27 | 11.92 | 11.93 | 11.97 | 11.91 |  |
| Malta |  |  |  |  |  |  |  |
| Netherlands | 11.76 | 11.91 | 12.13 | 12.23 |  |  | professionally active |
| Norway | 16.13 | 16.40 | 16.53 | 16.67 | 16.89 | 17.33 |  |
| Poland | 5.28 | 5.28 |  | 5.27 | 5.24 | 5.20 |  |
| Portugal | 5.68 | 5.88 | 5.99 | 6.05 | 6.13 | 6.29 | professionally active |
| Romania |  |  |  |  |  |  |  |
| Slovakia | 6.07 | 5.94 | 5.82 | 5.75 | 5.75 | 5.70 | professionally active |
| Slovenia | 8.19 | 8.33 | 8.16 | 8.32 | 8.56 | 8.78 |  |
| Spain | 5.15 | 5.22 | 5.24 | 5.14 | 5.15 | 5.29 |  |
| Sweden | 11.02 | 11.05 | 11.07 | 11.09 | 11.06 | 11.06 |  |
| United Kingdom | 8.41 | 8.20 | 7.94 | 7.94 | 7.94 | 7.91 |  |

**Supplementary Table S3d. Practising/ professionally active nurses per 1000 population, alternative data processing method (alt_nurses, missing values from OECD database complemented with Eurostat data) and its corresponding quotient of intEAPD and alt_nurses in Model M.18S). The variables nurses (Supplementary Table S3a) and alt_nurses showed strong significant correlation r_s_ = 0.879, p = 2*E-10).**

| **Country** | **alt_nurses** | **int_alt_EALOS** |
| --- | --- | --- |
| Austria | 7.87 | 0.69 |
| Belgium | 10.19 | 1.11 |
| Bulgaria | 4.36 | 0.10 |
| Croatia | 1.09 | 0.05 |
| Cyprus | 4.72 | 0.27 |
| Czechia | 8.02 | 0.37 |
| Denmark | 16.38 | 1.86 |
| Estonia | 5.99 | 0.55 |
| Finland | 14.12 | 0.97 |
| France | 9.20 | 1.03 |
| Germany | 12.15 | 0.94 |
| Greece | 3.33 | 0.04 |
| Hungary | 6.34 | 0.24 |
| Iceland | 15.13 | 0.16 |
| Ireland | 12.74 | 1.63 |
| Italy | 6.26 | 0.43 |
| Latvia | 4.87 | 0.25 |
| Lithuania | 7.55 | 0.59 |
| Luxembourg | 11.68 | 1.04 |
| Malta | 7.12 | 0.60 |
| Netherlands | 12.01 | 1.52 |
| Norway | 16.66 | 2.38 |
| Poland | 5.25 | 0.33 |
| Portugal | 6.00 | 0.47 |
| Romania | 0.45 | 0.01 |
| Slovakia | 5.84 | 0.29 |
| Slovenia | 8.39 | 0.87 |
| Spain | 5.20 | 0.52 |
| Sweden | 11.06 | 1.36 |
| United Kingdom | 8.06 | 0.77 |

**Supplementary Table S4. Comparison of the interpretation of the change in CRGN (ΔCRGN) due to changes in nurses/intEAPD/intEAlos between log-linear (M1.S, M2.S, M3.S and M19.S; untransformed Acpr) and respective log-log models (M1.M, M2.M, M3.M and M4.M; log-transformed Acpr). Overlapping 95% CI indicate no statistical difference between the respective pair of interpretations. Additionally, int_alt_EALOS was interpreted and compared to M3.S and M3.M with no statistically different interpretations observed at 5% significance level.**

| **Model** | **ΔCRGN** | **95% CI** | | |
| --- | --- | --- | --- | --- |
| M1.S | 8.3% | 1.0% | - | 16.2% |
| M1.M | 8.9% | 2.2% | - | 15.9% |
| M2.S | 13.3% | 4.7% | - | 22.6% |
| M2.M | 12.0% | 3.7% | - | 21.0% |
| M3.S | 0.4% | 0.1% | - | 0.7% |
| M3.M | 0.4% | 0.1% | - | 0.6% |
| M18.S | 0.4% | 0.1% | - | 0.7% |
| M4.M | 0.51% | 0.01% | - | 1.02% |
| M19.S | 0.58% | 0.04% | - | 1.13% |

**Supplementary Table S5. Assumption (a). Durbin-Watson (DW) statistic. Acceptable DW values are 1.5 - 2.5.**

| **DW statistic** | |
| --- | --- |
| M1.S | 1.99 |
| M1.M | 1.94 |
| M2.S | 1.82 |
| M2.M | 1.77 |
| M3.S | 1.96 |
| M3.M | 1.89 |
| M4.M | 1.77 |

**Supplementary Table S6. Assumption (b). Variance inflation factor (VIF). Acceptable values are between 1 and 3, while VIF values > 10 are cause of concern.**

| **Models** | **M1.S** | **M1.M** | **M2.S** | **M2.M** | **M3.S** | **M3.M** | **M4.M** |
| --- | --- | --- | --- | --- | --- | --- | --- |
| Acpr/log_Acpr | 1.97 | 2.31 | 2.50 | 3.09 | 2.13 | 2.61 | 2.81 |
| DDD | 1.25 | 1.23 | 1.27 | 1.25 | 1.26 | 1.24 | 1.22 |
| nurses/intEAPD/intEAlos/intEAlos_H | 2.85 | 2.82 | 3.53 | 3.67 | 2.36 | 2.42 | 2.13 |
| CPI | 3.25 | 3.60 | 2.89 | 3.00 | 2.50 | 2.72 | 2.39 |

**Supplementary Table S7. Cook’s distance (D). Rule of a thumb, cases with D > 3 * arithmetic mean (red cells) are potential influential outliers, while cases with D > 1 are a source of concern.**

| **Country** | **M1.S** | **M1.M** | **M2.S** | **M2.M** | **M3.S** | **M3.M** | **M4.M** |
| --- | --- | --- | --- | --- | --- | --- | --- |
| Austria | 0.008 | 0.027 | 0.008 | 0.025 | 0.008 | 0.027 | 0.053 |
| Belgium | 0.013 | 0.018 | 0.012 | 0.007 | 0.014 | 0.008 | 0.019 |
| Bulgaria | 0.432 | 0.019 | 0.406 | 0.023 | 0.438 | 0.027 | 0.061 |
| Croatia | 0.001 | 0.021 | 0.000 | 0.004 | 0.001 | 0.002 | 0.008 |
| Cyprus | 0.053 | 0.033 | 0.047 | 0.035 | 0.060 | 0.047 | 0.083 |
| Czech Republic | 0.079 | 0.080 | 0.133 | 0.136 | 0.156 | 0.161 |  |
| Denmark | 0.012 | 0.025 | 0.013 | 0.017 | 0.003 | 0.006 | 0.006 |
| Estonia | 0.001 | 0.095 | 0.001 | 0.073 | 0.000 | 0.094 | 0.096 |
| Finland | 0.002 | 0.005 | 0.000 | 0.008 | 0.028 | 0.000 | 0.045 |
| France | 0.015 | 0.000 | 0.009 | 0.000 | 0.022 | 0.000 | 0.000 |
| Germany | 0.027 | 0.010 | 0.046 | 0.030 | 0.015 | 0.003 | 0.006 |
| Greece | 0.067 | 0.013 | 0.018 | 0.029 | 0.028 | 0.031 | 0.006 |
| Hungary | 0.044 | 0.037 | 0.050 | 0.043 | 0.040 | 0.032 | 0.026 |
| Iceland | 0.102 | 0.037 | 0.026 | 0.129 | 0.003 | 0.016 | 0.036 |
| Ireland | 0.079 | 0.172 | 0.061 | 0.200 | 0.069 | 0.207 | 0.103 |
| Italy | 0.054 | 0.013 | 0.043 | 0.009 | 0.038 | 0.005 | 0.000 |
| Latvia | 0.003 | 0.000 | 0.008 | 0.004 | 0.004 | 0.001 | 0.000 |
| Lithuania | 0.022 | 0.003 | 0.023 | 0.006 | 0.013 | 0.001 | 0.000 |
| Luxembourg | 0.000 | 0.000 | 0.000 | 0.000 | 0.009 | 0.010 | 0.004 |
| Malta | 0.001 | 0.004 | 0.003 | 0.007 | 0.004 | 0.008 | 0.013 |
| Netherlands | 0.052 | 0.047 | 0.037 | 0.035 | 0.036 | 0.033 | 0.066 |
| Norway | 0.007 | 0.081 | 0.064 | 0.104 | 0.326 | 0.496 | 0.193 |
| Poland | 0.009 | 0.001 | 0.008 | 0.001 | 0.009 | 0.001 | 0.002 |
| Portugal | 0.044 | 0.014 | 0.048 | 0.015 | 0.046 | 0.014 | 0.014 |
| Romania | 0.044 | 0.011 | 0.006 | 0.000 | 0.005 | 0.001 | 0.001 |
| Slovak Republic | 0.004 | 0.000 | 0.001 | 0.001 | 0.000 | 0.002 |  |
| Slovenia | 0.031 | 0.058 | 0.029 | 0.034 | 0.004 | 0.008 | 0.021 |
| Spain | 0.001 | 0.021 | 0.000 | 0.014 | 0.002 | 0.024 | 0.011 |
| Sweden | 0.001 | 0.019 | 0.006 | 0.027 | 0.008 | 0.035 |  |
| United Kingdom | 0.151 | 0.240 | 0.130 | 0.145 | 0.101 | 0.129 | 0.054 |

**Additional References**

1 European Centre for Disease Prevention and Control. Antimicrobial resistance in the EU/EEA (EARS-Net) - Annual Epidemiological Report for 2016. 2017; published online Nov 15. https://www.ecdc.europa.eu/en/publications-data/antimicrobial-resistance-surveillance-europe-2016 (accessed Feb 20, 2019).

2 McDonnell L, Armstrong D, Ashworth M, Dregan A, Malik U, White P. National disparities in the relationship between antimicrobial resistance and antimicrobial consumption in Europe: an observational study in 29 countries. *J Antimicrob Chemother* 2017; **72**: 3199–204.

3 International Labour Office (ILO). International Standard Classification of Occupations (ISCO-08) 2012. https://www.ilo.org/wcmsp5/groups/public/---dgreports/---dcomm/---publ/documents/publication/wcms_172572.pdf (accessed November 19, 2021).

4 World Health Organization. Classifying health workers: Mapping occupations to the international standard classification 2010. https://www.who.int/hrh/statistics/Health_workers_classification.pdf (accessed November 18, 2021).

5 European Commission. Healthcare personnel statistics - nursing and caring professionals. Eurostat 2021. https://ec.europa.eu/eurostat/statistics-explained/index.php?title=Healthcare_personnel_statistics_-_nursing_and_caring_professionals (accessed November 18, 2021).

6 OECD. Health resources - Nurses - OECD Data. http://data.oecd.org/healthres/nurses.htm (accessed Aug 11, 2018).

7 Contact Point for Cross-Border Healthcare. Healthcare system in Finland. https://www.eu-healthcare.fi/healthcare-in-finland/healthcare-system-in-finland/ (accessed Jan 24, 2022).

8 Kankaanranta T, Rissanen P. Nurses’ Intentions to Leave Nursing in Finland. *Eur J Health Econ* 2008; **9**: 333–42.

9 Kaba HEJ, Kuhlmann E, Scheithauer S. Thinking outside the box: Association of antimicrobial resistance with climate warming in Europe – A 30 country observational study. *Int J Hyg Environ Health* 2020; **223**: 151–8.

10 Field A. Discovering statistics using IBM SPSS Statistics: and sex and drugs and rock ‘n’ roll, 4th edition. London: Sage, 2013.

11 Berry WD. Understanding Regression Assumptions. Newbury Park, Calif.: SAGE Publications, 1993.

12 Baron R, Kenny D. The moderator-mediator variable distinction in social psychological research: Conceptual, strategic, and statistical considerations. *J Pers Soc Psychol* 1986; **51**: 1173–82.

13 World Health Organization. Central Asian and European Surveillance of Antimicrobial Resistance (CAESAR). 2018 https://www.euro.who.int/en/health-topics/disease-prevention/antimicrobial-resistance/surveillance/central-asian-and-european-surveillance-of-antimicrobial-resistance-caesar (accessed Aug 11, 2018).

14 World Health Organization. Antimicrobial Medicines Consumption (AMC) Network. AMC data 2011–2014 (2017). 2017 https://www.euro.who.int/en/health-topics/Health-systems/health-technologies-and-medicines/publications/2017/antimicrobial-medicines-consumption-amc-network.-amc-data-20112014-2017 (accessed Aug 11, 2018).

15 Bundesamt für Gesundheit. Swiss Antibiotic Resistance Report 2016. https://www.bag.admin.ch/bag/de/home/das-bag/publikationen/broschueren/publikationen-uebertragbare-krankheiten/publikation-swiss-antibiotic-resistance-report-2016.html.

16 European Centre for Disease Prevention and Control. Antimicrobial consumption database (ESAC-Net). Eur. Cent. Dis. Prev. Control. https://www.ecdc.europa.eu/en/antimicrobial-consumption/surveillance-and-disease-data/database (accessed March 26, 2018).

17 European Commission. Eurostat Data Explorer - Nursing and caring professionals [HLTH_RS_PRSNS]. https://appsso.eurostat.ec.europa.eu/nui/show.do?dataset=hlth_rs_prsns&lang=en (accessed Aug 11, 2018).

18 European Commission. Eurostat - Data Explorer - Population on 1 January by age and sex [demo_pjan]. https://appsso.eurostat.ec.europa.eu/nui/show.do?dataset=demo_pjan&lang=en (accessed Aug 11, 2018).

19 European Commission. Eurostat - Data Explorer - Physicians by sex and age (hlth_rs_phys). https://appsso.eurostat.ec.europa.eu/nui/show.do?dataset=hlth_rs_phys&lang=en (accessed Aug 11, 2018).

20 World Bank. Current health expenditure (% of GDP) | Data. https://data.worldbank.org/indicator/SH.XPD.CHEX.GD.ZS (accessed Sept 10, 2019).

21 European Commission. Eurostat - Data Explorer - Hospital beds by type of care [hlth_rs_bds]. https://appsso.eurostat.ec.europa.eu/nui/show.do?dataset=hlth_rs_bds&lang=en (accessed Sept 9, 2019).

22 World Health Organization. European Health Information Gateway - Total number of hospital beds. https://gateway.euro.who.int/en/indicators/hlthres_40-curative-acute-care-beds-total/ (accessed Sept 9, 2019).

23 Institute for Health Metrics and Evaluation. GBD Results Tool | GHDx. 2018. http://ghdx.healthdata.org/gbd-results-tool (accessed April 9, 2018).

24 European Commission. Eurostat Data Explorer - In-patient average length of stay (days) [hlth_co_inpst]. http://appsso.eurostat.ec.europa.eu/nui/show.do?dataset=hlth_co_inpst&lang=en (accessed June 1, 2019).

25 OECD. Health Care Utilisation : Hospital average length of stay by diagnostic categories. https://stats.oecd.org/index.aspx?queryid=30165 (accessed June 1, 2019).

26 OECD. Health Care Utilisation : Hospital discharges by diagnostic categories. https://stats.oecd.org/index.aspx?queryid=30165# (accessed June 5, 2019).

27 Hospital discharges by diagnosis, in-patients, total number [hlth_co_disch1]. http://appsso.eurostat.ec.europa.eu/nui/setupDownloads.do (accessed June 5, 2019).

28 Transparency International. Corruption Perceptions Index (CPI). Transparency.org. https://www.transparency.org/research/cpi/overview (accessed March 28, 2018).

29 World Bank. World Bank Climate Change Knowledge Portal. https://climateknowledgeportal.worldbank.org/ (accessed Aug 11, 2018).

30 European Commission. Eurostat Data Explorer - Health personnel employed in hospital [HLTH_RS_PRSHP1]. https://ec.europa.eu/eurostat/databrowser/view/HLTH_RS_PRSHP1__custom_1900386/default/table?lang=en (accessed January 13, 2022).
